# Genomic landscape of patients with *FLT3*-mutated acute myeloid leukemia (AML) treated within the CALGB 10603/RATIFY trial

**DOI:** 10.1038/s41375-022-01650-w

**Published:** 2022-08-03

**Authors:** Nikolaus Jahn, Ekaterina Jahn, Maral Saadati, Lars Bullinger, Richard A. Larson, Tiziana Ottone, Sergio Amadori, Thomas W. Prior, Joseph M. Brandwein, Frederick R. Appelbaum, Bruno C. Medeiros, Martin S. Tallman, Gerhard Ehninger, Michael Heuser, Arnold Ganser, Celine Pallaud, Insa Gathmann, Julia Krzykalla, Axel Benner, Clara D. Bloomfield, Christian Thiede, Richard M. Stone, Hartmut Döhner, Konstanze Döhner

**Affiliations:** 1 Department of Internal Medicine III, University Hospital of Ulm, Ulm, Germany; 2 Saadati Solutions, Ladenburg, Germany; 3 Department of Hematology, Oncology and Tumor Immunology, Charité University, Berlin, Germany; 4 Department of Medicine and Comprehensive Cancer Center, University of Chicago, Chicago, IL USA; 5 Department of Biomedicine and Prevention, University Tor Vergata, Rome, Italy; 6 Santa Lucia Foundation, Neuro-Oncohematology, I.R.C.C.S., Rome, Italy; 7 Case Western Reserve University, Cleveland, OH USA; 8 Department of Medicine, University of Alberta, Edmonton, AB Canada; 9 Clinical Research Division, Fred Hutchinson Cancer Research Center, Seattle, WA USA; 10 Division of Hematology, Stanford Comprehensive Cancer Center, Stanford University, Stanford, CA USA; 11 Division of Hematologic Malignancies, Leukemia Service, Memorial Sloan Kettering Cancer Center, New York, NY USA; 12 Medizinische Klinik und Poliklinik I, Universitätsklinikum Carl Gustav Carus der TU Dresden, Dresden, Germany; 13 Department of Hematology, Hemostasis, Oncology and Stem Cell Transplantation, Hannover Medical School, Hannover, Germany; 14 Novartis Pharmaceuticals, Basel, Switzerland; 15 Division of Biostatistics, German Cancer Research Center Heidelberg, Heidelberg, Germany; 16 The Ohio State University Comprehensive Cancer Center, Columbus, OH USA; 17 Department of Medical Oncology, Dana-Farber/Partners CancerCare, Boston, MA USA

**Keywords:** Acute myeloid leukaemia, Cancer genetics

## Abstract

The aim of this study was to characterize the mutational landscape of patients with *FLT3*-mutated acute myeloid leukemia (AML) treated within the randomized CALGB 10603/RATIFY trial evaluating intensive chemotherapy plus the multi-kinase inhibitor midostaurin versus placebo. We performed sequencing of 262 genes in 475 patients: mutations occurring concurrently with the *FLT3*-mutation were most frequent in *NPM1* (61%), *DNMT3A* (39%), *WT1* (21%), *TET2* (12%), *NRAS* (11%), *RUNX1* (11%), *PTPN11* (10%), and *ASXL1* (8%) genes. To assess effects of clinical and genetic features and their possible interactions, we fitted random survival forests and interpreted the resulting variable importance. Highest prognostic impact was found for *WT1* and *NPM1* mutations, followed by white blood cell count, *FLT3* mutation type (internal tandem duplications vs. tyrosine kinase domain mutations), treatment (midostaurin vs. placebo), *ASXL1* mutation, and ECOG performance status. When evaluating two-fold variable combinations the most striking effects were found for *WT1*:*NPM1* (with *NPM1* mutation abrogating the negative effect of *WT1* mutation), and for *WT1*:treatment (with midostaurin exerting a beneficial effect in *WT1*-mutated AML). This targeted gene sequencing study provides important, novel insights into the genomic background of *FLT3*-mutated AML including the prognostic impact of co-mutations, specific gene–gene interactions, and possible treatment effects of midostaurin.

## Introduction

Mutations of *FLT3* (*fms-related tyrosine kinase 3*) are common gene mutations in acute myeloid leukemia (AML). They are found in 10–30% of patients, with a decreasing prevalence with older age [1, 2]. *FLT3* encodes a receptor-tyrosine kinase that is expressed on hematopoietic stem and progenitor cells and regulates cellular proliferation and apoptosis through downstream signaling pathways such as STAT5, RAS/MAPK, and PI3K/Akt [3]. Mutations of *FLT3* encompass internal tandem duplications (ITD), most frequently located in the juxtamembrane region, as well as point mutations in the tyrosine kinase domain (TKD), both promoting ligand-independent, constitutive receptor activation [4]. ITD but not TKD mutations have been consistently linked to a higher relapse rate and an inferior overall survival [5–7]. Yet, the prognostic impact of *FLT3*-ITD is context-dependent and influenced by the mutational background, for example whether a concurrent *NPM1* mutation is present or not, the location of the ITD insertion site, and the mutant to wildtype allelic ratio (AR) [5, 8–14]. The prognostic significance of the *NPM1* mutational status and of the *FLT3*-ITD AR is implemented in the 2017 European LeukemiaNet (ELN) risk stratification [15].

The successful development of first- and next-generation FLT3 inhibitors has contributed to a new era for precision medicine in AML [16]. In the international CALGB 10603/RATIFY trial, the addition of the oral multi-targeted small molecule *FLT3* inhibitor midostaurin to intensive induction and consolidation chemotherapy, followed by a 1-year midostaurin maintenance therapy, improved overall survival of patients 18 to 59 years of age with newly diagnosed *FLT3*-mutated AML [17]. Midostaurin showed consistent effects across all *FLT3* mutation strata, TKD, and ITD independent of the AR [17]. In addition, in the AMLSG 16–10 trial midostaurin significantly improved outcome also in older patients 60 to 70 years of age in comparison to a historical control cohort [18].

We previously reported on the impact of *NPM1*/*FLT3*-ITD genotypes as defined by 2017 ELN risk categories and of *FLT3*-TKD mutations in patients of the RATIFY trial [12, 19]. The objective of the current study was to comprehensively profile the mutational landscape of *FLT3*-mutated AML in patients treated within the RATIFY trial using a high-throughput targeted sequencing (HTS) approach, and to correlate genetic patterns with clinical outcomes.

## Patients and methods

### Patients

Overall, 717 patients with AML and *FLT3* mutation were enrolled in the CALGB 10603/RATIFY trial [17]. HTS was performed on 475 patients (*n* = 409 bone marrow, *n* = 66 peripheral blood) who gave informed consent for further molecular studies and for whom DNA was available (Supplementary Table S1). Patient characteristics are provided in Table 1. *FLT3* mutation status was available for all 475 pts (TKD: *n* = 116; 24%; ITD: *n* = 335; 71%; TKD + ITD: *n* = 24; 5%) and information on the AR (0.05–0.5: *n* = 126; ≥0.5: *n* = 232) for all but one patient with ITD. For analyses, patients (*n* = 24) harboring both *FLT3*-TKD and -ITD mutations were assigned to the ITD group based on the prognosis-defining features of ITD compared to TKD mutations.

**Table 1:** Baseline characteristics of 475 patients with *FLT3*-mutation.

	Placebo (*N* = 222)	Midostaurin (*N* = 253)	Overall (*N* = 475)
Age at registration (years)			
Mean (SD)	46.0 (11.3)	45.2 (10.6)	45.5 (10.9)
Median [Min, Max]	49.3 [18.0, 59.9]	47.1 [19.0, 59.8]	47.9 [18.0, 59.9]
Sex			
Male	90 (40.5%)	118 (46.6%)	208 (43.8%)
Female	132 (59.5%)	135 (53.4%)	267 (56.2%)
*FLT3* mutation type			
TKD	54 (24.3%)	62 (24.5%)	116 (24.4%)
ITD < 0.5 allelic ratio	61 (27.5%)	65 (25.7%)	126 (26.5%)
ITD ≥ 0.5 allelic ratio	106 (47.7%)	126 (49.8%)	232 (48.8%)
Missing	1 (0.5%)	0 (0%)	1 (0.2%)
WBC count at baseline (10E9/L)			
Mean (SD)	54.1 (58.8)	48.7 (46.6)	51.2 (52.7)
Median [Min, Max]	33.1 [0.8, 330]	36.4 [0.6, 304]	35.4 [0.6, 330]
Missing	1 (0.5%)	2 (0.8%)	3 (0.6%)
ECOG performance status			
0–1	193 (86.9%)	229 (90.5%)	422 (88.8%)
2	29 (13.1%)	24 (9.5%)	53 (11.2%)
Treatment			
Placebo	222 (100%)	0 (0%)	222 (46.7%)
Midostaurin	0 (0%)	253 (100%)	253 (53.3%)

*ECOG* Eastern Cooperative Oncology Group, *WBC* white blood cell count, *SD* standard deviation.

In the RATIFY trial, patients 18 to 59 years of age were randomly assigned to receive standard chemotherapy plus either midostaurin or placebo. Randomization was stratified according to the subtype of *FLT3* mutation: TKD or ITD with either a low (0.05 to 0.7) or a high AR (>0.7). For this analysis, a threshold of 0.5 (low, 0.05 to <0.5; high, ≥0.5) was chosen as this cutoff has been demonstrated to better discriminate clinical outcome and was adopted in the 2017 ELN risk classification [15]. 253 (53%) of 475 patients were randomized to the midostaurin arm and 222 patients to the placebo arm. 128 (27%) of the patients underwent allogeneic hematopoietic cell transplantation (HCT) in first complete remission (CR).

The study was approved by the institutional review board at each participating center. Written consent was given by all patients for treatment, genetic analysis, and biobanking according to the Declaration of Helsinki.

### Gene mutation analyses

HTS was performed on the entire coding region of 262 genes involved in hematologic malignancies including 20 kinases targeted by midostaurin [20]. A comprehensive list of all genes is included in the Supplementary Appendix (Supplementary Tables S2, S3). Library enrichment was performed using SureSelectXT from Agilent Technologies (Santa Clara, CA, USA). Paired-end sequencing (read length: 2 × 100 base pairs) was carried out on a HiSeq 2000 platform (Illumina, San Diego, CA, USA). All sequencing data were analyzed using an in-house computational pipeline [21]. Detailed information on the mutation calling and data curation workflow is provided in the Supplementary Information. *FLT3* mutations were assessed by GeneScan-based fragment-length analysis as previously described [17].

### Statistical analyses

Statistical analyses were performed using R, version 3.6.3. Fisher’s exact test and chi-squared test were employed for two-group comparisons of categorical variables and the Wilcoxon rank-sum test for continuous variables. The Kaplan–Meier method was used to estimate the distributions of time-to-event endpoints event-free survival (EFS) and overall survival (OS). Associations between clinical and genetic variables and time-to-event endpoints were analyzed using log rank test and random survival forests.

Additionally, for the analysis of genomic classes log rank tests and multivariable Cox proportional hazards regression models were used, for which the proportional hazards assumption was checked via graphical methods. The covariables age, gender, ECOG performance status (0–1 vs. 2), WBC count on log2 scale, treatment arm, allogeneic HCT in first remission (as time-dependent variable), *FLT3*-mutation status as well the genomic classes (*NPM1*, chromatin-spliceosome, no class, *TP53-*aneuploidy, and core-binding factor (CBF) AML) were introduced into the multivariable regression model.

An effect was considered significant if *P* ≤ 0.05. As the proportion of missing values in covariates used for modeling was very low (1 missing observation in *FLT3* allelic ratio, 3 missings in white blood cell count [WBC]), single imputation was employed using chained equations as implemented in the mice package [22]. Clinical endpoints were assessed as defined by the 2017 ELN recommendations [15, 17].

Correction for multiple testing was implemented via the Bonferroni-Holm and the Benjamini–Hochberg method, depending on the number of tests [23, 24]. An effect was considered significant if the adjusted *P* ≤ 0.05.

Mutual exclusivity and co-occurrence of genomic mutations were tested via independence tests while accounting for varying mutation rates via the method proposed by Canisius et al. and implemented in the Rediscover R package [25].

Random survival forests were implemented using the R package randomForestSRC to assess possible interactions of clinical and genetic variables [26]. One-hundred forests with distinct seeds were fitted on the imputed dataset hereby growing 1000 trees for each forest. Tree nodes were split using log rank statistics. The impact of a variable was ranked by the variable importance (VIMP) based on the prediction error calculated using Harrell’s C-index [27]. The assessment of pairwise interactions between variables was based on the comparison of the joint (“paired”) VIMP to the sum of their individual VIMPs (called “additive” importance): A large positive or negative difference between “additive” and “paired” importance indicates a possibly relevant association.

## Results

### Mutational landscape of *FLT3*-mutated AML

**Fig. 1:**
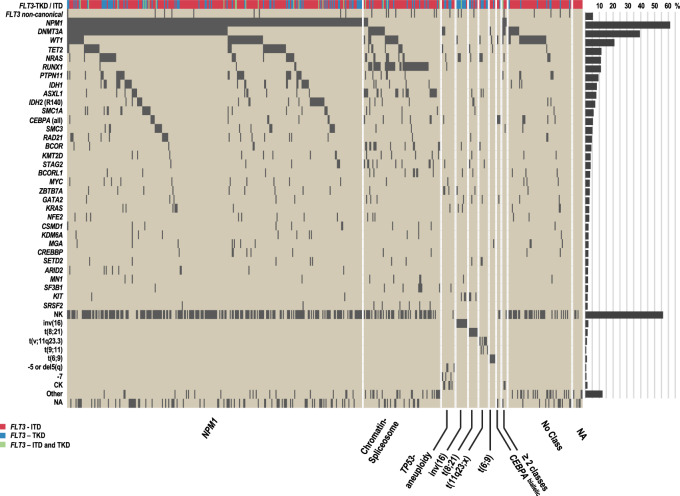
Frequency of recurrently mutated genes as well as cytogenetics in 475 *FLT3*-mutated patients categorized according to recently defined genomic classes [1]. Each column represents a single patient, each dark gray colored box indicates a specified driver mutation or cytogenetic feature. Wildtype cases are illustrated in beige, *FLT3*-ITD mutations in dark red, *FLT3*-TKD mutations in blue. Bar plots indicate the relative frequency of all aberrations in the entire cohort. CK complex karyotype, NA not available, NK normal karyotype.

**Fig. 2:**
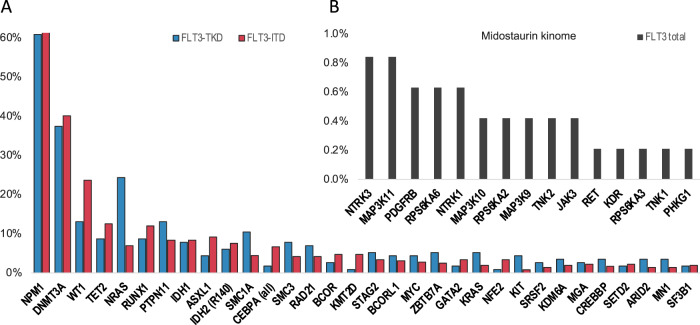
Mutational landscape of *FLT3*-mutated acute myeloid leukemia. **A** Mutational landscape of 475 patients stratified according to the *FLT3* mutation type. **B** Incidence of mutations in the midostaurin kinome in the entire cohort of 475 patients.

High-throughput sequencing of the 475 *FLT3*-mutated AML yielded a mean on-target sequencing depth of ~975×. Overall, we found 1988 mutations with a variant allele fraction of equal to or greater than 3% in 200 of the 262 genes. At least one mutation in addition to *FLT3*-ITD or -TKD was identified in 463 of 475 (97.5%) patients. The overall median number of additional mutations per patient was 3 and—when stratified by mutation type—4 in the TKD vs. 3 in the ITD group. The majority of mutations were missense mutations (49%) and indels (40%). 26 (5.5%) of patients had non-canonical *FLT3* mutations outside of TKD and ITD mutational hotspots, including 14 with mutations in exon 16 (codon 663–680), that were missed by GeneScan-based fragment-length analysis. 4 of 14 were concurrent with ITDlow and 10 of them with TKD mutations. Overall, the most frequently affected genes were *NPM1* (291, 61%), *DNMT3A* (187, 39%), *WT1* (100, 21%), *TET2* (55, 12%), *NRAS* (53, 11%), *RUNX1* (53, 11%), *PTPN11* (45, 10%), *IDH1* (39, 8%), *ASXL1* (38, 8%), *IDH2* (34, 7%), *SMC1A* (28, 6%), *CEBPA* (26, 5%), and *SMC3* (24, 5%) (Figs. 1, 2; Table S4).

#### Frequency of gene mutations by FLT3 mutation type

*NRAS* (28, 24% vs. 25, 7%, *p* < 0.001), *SMC1A* (12, 10% vs. 16, 4%, *p* = 0.02), and *KIT* (5, 4% vs. 3, 1%, *p* = 0.02) mutations occurred significantly more often in TKD than ITD groups, respectively, whereas *WT1* (15, 13% vs. 84, 24%, *p* = 0.018) was more frequently co-mutated in the ITD group (Fig. 2A, Table S4).

#### Frequency of mutations by functional classes (signaling, methylation, chromatin, cohesin, transcription, tumor suppressor, splicing, other)

In 29% (138) of cases we identified mutations affecting other signaling molecules, most commonly in *NRAS* (53, 11%), *PTPN11* (45, 10%), *KRAS* (13, 3%), and *KIT* (8, 2%) (categorization in Table S5). These additional signaling mutations more often co-occurred in the *FLT3*-TKD than -ITD group (50, 43% vs. 90, 25%, *p* = 0.001). In general, mutations altering DNA methylation pathways were common (total: 264, 56%; *DNMT3A*: 187, 39%, *TET2*: 55, 12%, *IDH1*: 39, 8%, *IDH2*^R140^: 34, 7%). Other frequently altered functional groups of genes involved chromatin modification (*ASXL1*: 38, 8%, *KMT2D*: 18, 4%) and the cohesin complex (*SMC1A*: 28, 6%, *SMC3*: 24, 5%, *RAD21*: 23, 5%, *STAG2*: 18, 4%). Among genes regulating transcription, we found recurrent loss-of-function mutations in *NFE2* (13, 3%) and codon 73–78 hotspot mutations in *MYC* (15, 3%), as well as aberrations of *WT1* (100, 21%), *RUNX1* (53, 11%), *CEBPA* (26, 5%), *BCOR* (20, 4%), *BCORL1* (16, 3%), *ZBTB7A* (15, 3%), and *GATA2* (14, 3%). Mutations in *TP53* (4, 0.8%) and in genes encoding for splicing factors (*SF3B1*: 9, 1.9%, *SRSF2*: 8, 1.7%, *U2AF1*: 4, 0.8%, *ZRSR2*: 1, 0.2%) were rare reflecting the de novo disease onset in the vast majority of cases (455, 96%) and the younger age of the patients.

The top 12 most frequently mutated genes were tested for mutual exclusivity (66 possible combinations) resulting in 21 significant pairs before adjustment for multiple testing and 5 significant pairs after FDR adjustment: *NPM1*-*RUNX1* (*p* < 0.001), *DNMT3A*-*WT1* (*p* < 0.001), *DNMT3A*-*RUNX1* (*p* = 0.003), *NPM1*-*WT1* (*p* = 0.008) and *IDH2*-*TET2* (*p* = 0.021). Similarly, all combinations were tested for co-occurrence resulting in 2 significant pairs before adjustment for multiple testing: *NPM1*-*DNMT3A* and *IDH1*-*PTPN11*, which were not significant after FDR adjustment (Fig. S1).

#### Classification of cases according to genomic AML classes

Using the mutational and cytogenetic data, we assigned cases to the genomic AML classes as previously defined (Fig. 1; Tables S6, 7) [1]. This was possible for 451 of 475 patients, in which the complete mutational and cytogenetic dataset was available or at least one class-defining aberration was present. The majority of cases fell into three classes: *NPM1* (287, 60%), chromatin-spliceosome (68, 14%), and no class (60, 13%). All other AML classes were less frequent: *TP53*-aneuploidy (18, 4%), inv(16) (10, 2%), t(8;21) (8, 2%), t(11q23;x) (8, 2%), t(6;9) (4, 1%), and *CEBPA*^biallelic^ (3, <1%). Due to these low sample sizes, we restricted further statistical analyses to the following genomic classes: *NPM1*, chromatin-spliceosome, no class, *TP53-*aneuploidy, and the core-binding factor (CBF) AML class [inv(16) and t(8;21)], thereby excluding a total of 24 patients in the smaller categories resulting in a total of 451 patients for analysis.

#### Mutations of midostaurin kinome genes

In addition, we screened 20 kinase genes (Table S3) known to be pharmaceutically targeted by midostaurin. Pretreatment mutations in the midostaurin kinome were rare events with only 40 mutations in 35 of 475 patients (7%). The most frequently affected genes were *KIT* (8, 2%), *NTRK3* (4, 0.8%), and *MAP3K11* (4, 0.8%) (Fig. 2B).

### Clinical impact of molecular landscape in *FLT3*-mutated AML

#### Prognostic and predictive impact of concurrent gene mutations

We first performed exploratory unvariable analysis for genes that were mutated in more than 5% of 475 cases. Five of twelve mutations showed an association with outcome: mutations in *ASXL1* (*p* = 0.0125; hazard ratio (HR): 1.67, 95% confidence interval (^95%^CI): 1.11–2.52) and *WT1* (*p* < 0.001; HR: 1.83, ^95%^CI: 1.38–2.43) were associated with a significantly shortened OS (Table S8 and Figs. S2, 3), whereas *NPM1* (*p* < 0.001; HR: 0.6, ^95%^CI: 0.46–0.77) and in trend *NRAS* (*p* = 0.097; HR: 0.68, ^95%^CI: 0.43–1.08) mutations conferred a favorable prognosis. After adjusting for multiple testing via Bonferroni-Holm procedure, only *NPM1* and *WT1* mutations retained significance at the 5% level. With respect to EFS, *WT1* (*p* = 0.018; HR: 1.36, ^95%^CI: 1.05–1.75), and in trend *SMC1A* (*p* = 0.075; HR: 0.64, ^95%^CI: 0.38–1.05) conferred poor prognosis in univariate analysis, whereas only *NPM1* (*p* < 0.001; HR: 0.6, ^95%^CI: 0.48–0.74) prolonged survival, both in univariate analysis and after adjustment for multiple testing (Table S8, and Figs. S2, 3).

**Fig. 3:**
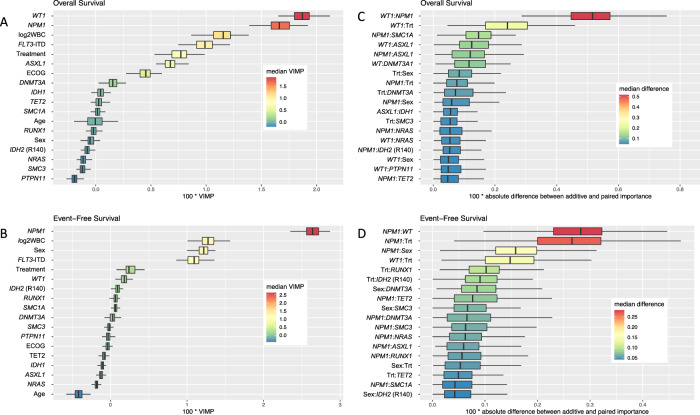
Random survival forest model for impact of clinical and/or genetic variables as well as pairwise interactions on prognosis. Prognostic and potential predictive impact of 18 most relevant clinical and/or genetic variables (**A**, **B**) and pairwise interactions (**C**, **D**) in 475 patients on overall and event-free survival using random survival forests. The prognostic impact of a variable is measured via VIMP (variable importance). Higher VIMP values indicate that a variable may have prognostic impact on the survival endpoint. EFS event-free survival, OS overall survival.

To assess prognostic effects of both clinical features and genetic variables we fitted random survival forests. To quantify the random variability, a total of 100 forests with different seeds were trained hereby constructing 1000 trees for each forest. The impact of a variable was measured using the variable importance (VIMP) in each forest which reflects the loss in the model’s prediction accuracy after permutation of each variable/feature. The higher the VIMP, the higher the prognostic and/or predictive value of the variable. With respect to OS, highest VIMP was found for *WT1* and *NPM1* mutations, followed by WBC count, *FLT3* mutation type, treatment (midostaurin vs. placebo), *ASXL1* mutation, and ECOG performance status (0–1 vs. 2) in descending order (Fig. 3A). With regard to EFS, random forest indicated a higher VIMP only for *NPM1*, WBC count, sex, and *FLT3* mutation status (Fig. 3B). The unfavorable impact of *WT1* mutations on OS was predominantly observed across the intermediate and adverse ELN 2017 groups (Figs. S4, 5).

**Fig. 4:**
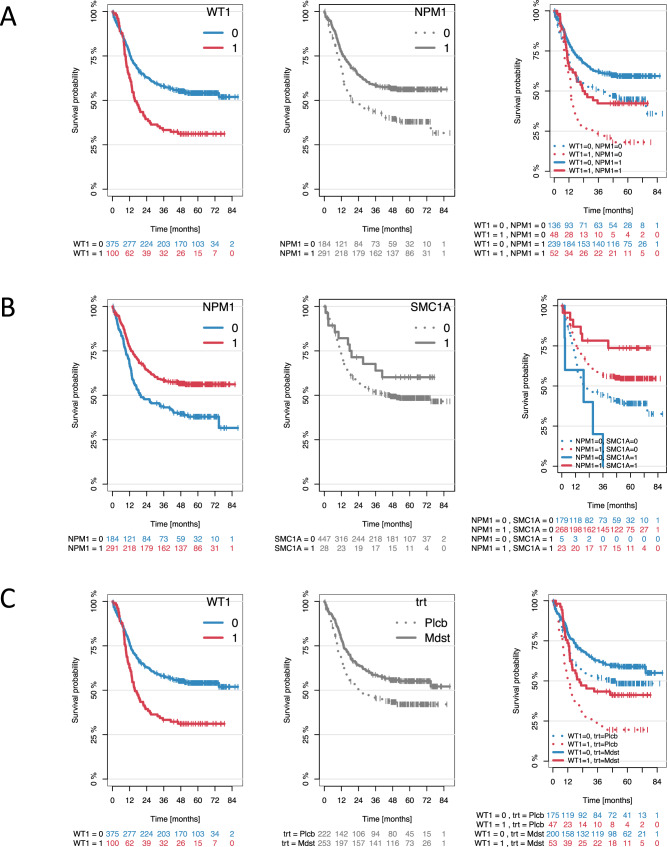
The three most interesting interactions regarding overall survival as selected by random survival forests. *NMP1*:*WT1* (**A**), *NPM1*:*SMC1A* (**B**) and *WT1*:treatment (**C**). Kaplan–Meier plots illustrating the influence of the clinical and/or genetic variables and their combination on overall survival: first variable (left) and the second variable (middle) as well as the combination of the two (right).

To assess the potential impact of specific gene–gene or gene-clinical variable interactions, resulting random forests were also used to identify relevant pairwise interactions among all paired combinations of mutations, midostaurin treatment, and gender. With regard to OS, the most striking interactions were observed for *WT1*:*NPM1* and *WT1*:treatment (Figs. 3C, S6). For the ten most relevant interactions we created Kaplan–Meier estimators for further illustration (Figs. 4, S7, 8). There were two important observations: first, the prognostic influence of some gene mutations was severely dependent on the mutational background: As aforementioned, outcome of patients harboring *NPM1* mutations was particularly favorable, whereas *WT1* mutations were linked to poor prognosis. In case of mutational co-occurrence, the presence of *NPM1* seemed to extensively abrogate the negative impact of *WT1* (Fig. 4A). Patients who had both *NPM1* and *SMC1A* mutations had a particularly good outcome, outreaching the prognosis of patients with the *NPM1*^mut^/*SMC1A*^wt^ genotype. Yet, in the absence of *NPM1* aberrations, all 5 patients with *SMC1A*-mutated AML died within 3 years (Fig. 4B). Second, specific genotypes in *FLT3*-mutated AML seemed to benefit particularly from therapy with midostaurin, in particular those patients with concurrent *WT1* mutation. In contrast, *WT1-*mutated patients randomized to the placebo arm had a very poor prognosis suggesting a particular benefit of midostaurin in the presence of *WT1*-mutations (Fig. 4C).

Of note; in line with previous data, *FLT3* was frequently co-mutated with *DNMT3A* and *NPM1* (*n* = 157; 33%). [1, 28–30] In our cohort, no relevant interaction could be observed for the combined *FLT3*:*DNMT3A:NPM1* genotype with regard to clinical outcome (OS, EFS) (Figs. 3C, D, S9).

#### Prognostic impact of FLT3 mutations by AML genomic class

In 451 of 475 cases that could be subclassified into genomic classes, we found significant differences in longterm survival among the five genomic AML classes (OS: *p* < 0.001, EFS: *p* < 0.001) (Fig. S10, Tables S9, 10). Estimated 4-year-survival rates were highest for CBF-AML (OS: 72%, EFS: 50%) followed by *NPM1* classes (OS: 57%, EFS: 38%), whereas patients of the chromatin-spliceosome (OS: 33%, EFS: 16%), *TP53*-aneuploidy (OS: 35%, EFS: 6%), and no class (OS: 37%, EFS: 10%) groups had an adverse OS and EFS. Although patients of the ‘no class’ group were younger, they had a higher frequency of *FLT3*-ITD, in particular with a high ITD AR, *WT1* co-mutations, and less often a normal karyotype, possibly explaining the poorer outcome (Fig. 1, Table S7).

**Fig. 5:**
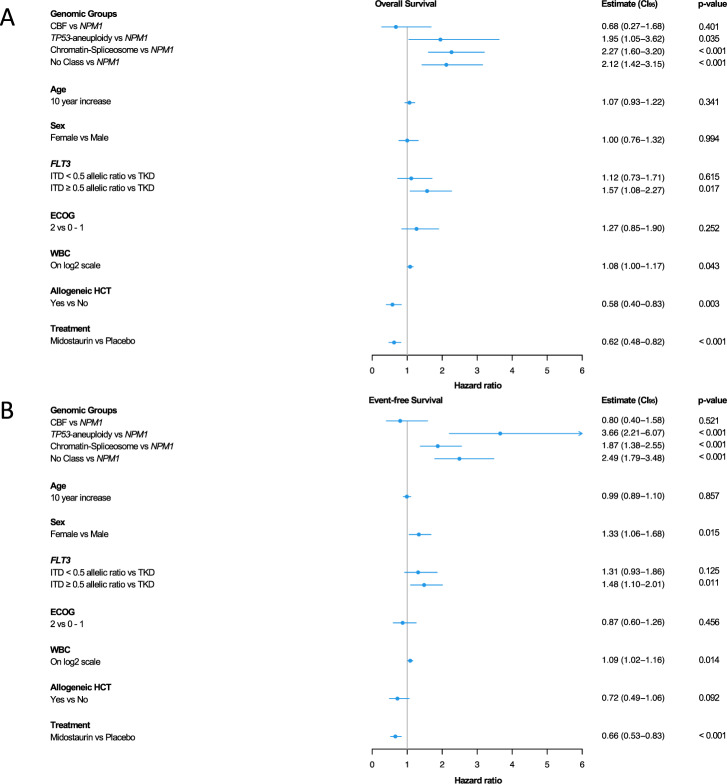
Prognostic impact of genomic AML classes and clinical characteristics in *FLT3*-mutated AML. Cox proportional hazard model for prognostic impact of genomic AML classes and clinical features on (**A**) overall and (**B**) event-free survival in the cohort of 451 of 475 patients, in which subcategorization into genomic AML classes was possible. A hazard ratio of >1 indicates a higher and a hazard ratio of <1 a lower hazard of death or event, respectively. The *NPM1* class was used as reference group, allogeneic HCT in first remission as time-dependent variable. ECOG Eastern Cooperative Oncology Group, HCT hematopoietic cell transplantation, WBC white blood cell count.

Next, we developed a prognostic proportional hazard model including the 5 genomic AML classes, as well as the covariates age, gender, ECOG performance status (0–1 vs. 2), log2 WBC count, *FLT3*-status (TKD, ITD^low^, ITD^high^), treatment (midostaurin, placebo), and allogeneic HCT in first CR1 as a time-dependent variable. In line with univariate analyses, we observed poorer OS and EFS in the *TP53-*aneuploidy, chromatin-spliceosome and no class genomic groups compared to the reference *NPM1* group (Fig. 5). CBF-AML patients seemed to have a more favorable outcome than the *NPM1* reference group, although not statistically significant likely due to the small sample size. With regard to the *FLT3*-status, patients with *FLT3*-ITD^high^ had inferior outcome compared to the *FLT3*-TKD mutated group. In addition, higher WBC counts conferred poor OS and EFS: A doubling was associated with an almost 10% higher risk of an event or death. Treatment with midostaurin and allogeneic HCT in CR1 both had a positive impact on OS, midostaurin in addition on EFS (Fig. 5).

Finally, we assessed whether the genomic classification had a predictive impact on the hazard of death after midostaurin treatment. Treatment with midostaurin prolonged OS in the *NPM1*, chromatin-spliceosome and in the no class groups (Table 2). This was not the case for *TP53*-aneuploidy and CBF-AML classes (Table 2, Figs. S11, 12). However, data need to be interpreted with caution due to the limited sample size. With regard to OS and EFS, similar treatment effects of midostaurin were observed across all *FLT3* mutation strata substantiating the therapeutic benefit irrespective of the *FLT3* mutation type (Table S11; Fig. S13).

**Table 2:** Cox proportional hazard model for predictive impact of genomic AML classes on hazard of death or event after treatment with midostaurin in the cohort of 451 of 475 patients, in which subcategorization into genomic AML classes was possible.

	Overall survival		Event-free survival	
Variable	HR [CI. 95]	*p* value	HR [CI. 95]	*p* value
Age	1.01 [0.99; 1.02]	0.357	1.00 [0.99; 1.01]	0.841
Sex				
Male	Reference			
Female	1.01 [0.77; 1.33]	0.940	1.34 [1.06; 1.69]	0.013
*FLT3* mutation type				
TKD	Reference			
ITD < 0.5 allelic ratio	1.15 [0.75; 1.76]	0.533	1.36 [0.96; 1.93]	0.085
ITD ≥ 0.5 allelic ratio	1.58 [1.09; 2.28]	0.016	1.52 [1.12; 2.05]	0.007
ECOG (0–1 vs. 2)	1.27 [0.84; 1.91]	0.261	0.90 [0.62; 1.31]	0.595
log2WBC	1.08 [1.00; 1.17]	0.046	1.08 [1.01; 1.16]	0.018
Allogeneic HCT in CR1	0.59 [0.41; 0.84]	0.004	0.71 [0.48; 1.04]	0.077
*NPM1*				
Midostaurin vs. Placebo	0.65 [0.45; 0.93]	0.018	0.76 [0.57; 1.03]	0.077
CBF				
Midostaurin vs. Placebo	1.37 [0.23; 8.23]	0.729	0.88 [0.24; 3.29]	0.853
*TP53*-aneuploidy				
Midostaurin vs. Placebo	1.11 [0.32; 3.86]	0.866	0.39 [0.15; 1.02]	0.055
Chromatin-Spliceosome				
Midostaurin vs. Placebo	0.52 [0.29; 0.92]	0.026	0.48 [0.28; 0.82]	0.007
No Class				
Midostaurin vs. Placebo	0.55 [0.28; 1.07]	0.079	0.64 [0.36; 1.13]	0.123
Placebo				
CBF vs. *NPM1*	0.46 [0.11; 1.92]	0.289	0.77 [0.31; 1.91]	0.569
* TP53*-aneuploidy vs. *NPM1*	1.42 [0.51; 3.93]	0.499	5.40 [2.58; 11.29]	<0.001
Chromatin-Spliceosome vs. *NPM1*	2.52 [1.58; 4.03]	<0.001	2.38 [1.55; 3.66]	<0.001
No Class vs. *NPM1*	2.31 [1.31; 4.05]	0.004	2.71 [1.66; 4.44]	<0.001
Midostaurin				
CBF vs. *NPM1*	0.99 [0.31; 3.20]	0.985	0.89 [0.32; 2.44]	0.816
* TP53*-aneuploidy vs. *NPM1*	2.45 [1.11; 5.42]	0.027	2.74 [1.37; 5.47]	0.004
Chromatin-Spliceosome vs. *NPM1*	2.02 [1.22; 3.34]	0.006	1.49 [0.96; 2.32]	0.073
No Class vs. *NPM1*	1.96 [1.14; 3.36]	0.014	2.28 [1.48; 3.51]	<0.001

A hazard ratio of >1 indicates a higher and a hazard ratio of <1 a lower risk of death or event, respectively.

*CBF* Core-binding factor AML, *CI. 95* 95% confidence interval, *CR1* first complete remission, *ECOG* Eastern Cooperative Oncology Group, *HR* hazard ratio, *HCT* hematopoietic cell transplantation, *WBC* white blood cell count.

## Discussion

In this study we characterized the mutational landscape and its clinical significance in 475 patients with *FLT3*-mutated AML enrolled on the pivotal CALGB 10603/RATIFY trial evaluating intensive chemotherapy with or without midostaurin.

The most frequent gene mutations co-occurring with *FLT3* mutations were, in order of decreasing frequency, mutations in *NPM1*, *DNMT3A*, *WT1*, *TET2*, *NRAS*, *RUNX1*, *PTPN11*, *IDH1*, *ASXL1*, *IDH2*, *SMC1A*, *CEBPA*, and *SMC3* genes. Compared to the general AML population, mutations in *WT1* were overrepresented which is in line with previous studies showing this particular co-mutation pattern [31, 32]. In contrast, mutations in *TP53*, and in genes encoding for splicing factors (*SF3B1*, *SRSF2*, *U2AF1*, *ZRSR2*) were less common, likely reflecting differences in disease biology—e.g., high frequency of normal karyotypes and *NPM1* mutations generally observed in *FLT3*-mutated AML—and age distribution (median age 47.9 years) compared to the median age of the general AML population of about 70 years [33].

To assess prognostic and potentially predictive effects of gene mutations in the context of clinical covariates, we fitted random forests. Here, the highest prognostic effect was found for *WT1* and *NPM1* mutations, followed by WBC count, *FLT3* mutation type (ITD vs. TKD mutations), treatment (midostaurin vs. placebo), *ASXL1* mutation, and ECOG performance status. The prognostic effect of *NPM1* mutations, especially in the context of concurrent *FLT3*-ITD, is well established and reflected in the 2017 ELN risk stratification [15]. The results from this study provide additional novel and clinically relevant data, in that mutations in *WT1* and *ASXL1* have independent prognostic value in the context of AML with *FLT3* mutations. In previous studies in pediatric AML and in cytogenetically-normal adult AML, the presence of *WT1* mutations has been linked to primary resistance to therapy and shortened survival [34–37]. Here, the unfavorable effect was most pronounced in the intermediate and adverse ELN risk group, whereas the effect in the favorable-risk group was largely mitigated by the concurrent presence of *NPM1* mutation. WT1 is a co-transcriptional factor involved in epigenetic regulation [38]. Several groups have reported that WT1 physically interacts with TET2 to modulate 5-hydroxymethylation of cytosine, a pathway commonly dysregulated in AML [39, 40]. Interestingly, in a recent study Pronier et al. have demonstrated that WT1 haploinsufficiency cooperates with *FLT3*-ITD to induce leukemogenesis in vivo [41].

Extending on the random forest model, when analyzing pairwise interactions the most striking interactions were observed for *WT1*:*NPM1* and *WT1*:treatment. The presence of *NPM1* mutations seemed to largely abrogate the negative impact of *WT1* mutations. A similar observation has previously been made for other *NPM1* co-mutation patterns, e.g., *NPM1*:*FLT3*-TKD, in that *NPM1* mutation abrogates the negative prognostic effect of a *FLT3*-TKD mutation [19, 42–44]. With regard to treatment effects, patients with *FLT3*:*WT1* co-mutations appeared to benefit from the addition of midostaurin; overall, patients with *FLT3*:*WT1* co-mutations had poor outcomes, however, patients with *FLT3*:*WT1* co-mutations on the midostaurin arm had similar longterm survival compared to patients with the *FLT3*^mut^/*WT1*^wt^ genotype treated on the placebo arm. Another new finding relates to the *FLT3*:*NPM1*:*SMC1A* gene–gene interaction. Patients who had co-mutation of both *NPM1* and *SMC1A* genes had a particularly good outcome, outreaching the prognosis of patients with the *FLT3*^mut^/*NPM1*^mut^/*SMC1A*^wt^ genotype. Yet, in the absence of *NPM1* mutations all patients harboring *a SMC1A* mutation died. Little is known about the biological basis of *SMC1A* mutations in the context of *NPM1* and *FLT3* mutations. However, AML with concurrent cohesin and *NPM1* gene mutations define a specific cluster within the group of *NPM1* mutated AML, that is reflected by a distinct metabolic gene signature [45]. Whether or not these features render the *NPM1*^mut^/*SMC1A*^mut^ genotype more susceptible to treatment with chemotherapy ± midostaurin remains to be determined. Another similar gene–gene interaction with a cohesin complex gene was previously found with the interaction of *NPM1* with *RAD21* mutations, i.e., AML with the *NPM1*^mut^/*RAD21*^mut^ genotype have been shown to be associated with high response rates and favorable longterm outcome [1]. Although these findings are very interesting and provide basis for further studies, they have to be interpreted cautiously due to small numbers.

In line with previous reports, the *FLT3:DNMT3A:NPM1* genotype represented a common co-mutational pattern in our study. Data on the impact of *FLT3:DNMT3A:NPM1* genotypes are still discussed controversially—with some publications showing evidence of poor outcome whereas others do not [1, 28–30]. Differences may be explained by variations in cohort size and features (e.g., age distribution), treatment, and genotypes (*DNMT3A* total vs. *DNMT3A* non-R882 vs. R882, *FLT3* total vs. *FLT3*-ITD). In our study the *FLT3:DNMT3A:NPM1* genotype did not impact clinical outcome. However, the analysis might be hampered by the size and heterogeneity of this specific subpopulation.

Based on the co-mutational and cytogenetic data we categorized the majority of the cases into four of the recently defined genomic AML classes [1] and evaluated whether the underlying class impacted response to therapy with midostaurin. A strong beneficial effect was seen for midostaurin treatment in the *NPM1*, chromatin-spliceosome and to a lesser extent “no class” groups. No impact of midostaurin was found for CBF- and *TP53*/aneuploidy groups which is likely attributable to the small group sizes. Overall, these results suggest a broad beneficial effect of midostaurin across various genetic backgrounds.

In conclusion, our targeted gene sequencing study in more than 450 patients with *FLT3* mutation entered on the randomized CALGB 10603/RATIFY trial provides important and novel insights into the genomic background of *FLT3*-mutated AML, on prognostic impact of mutations co-occurring with *FLT3* mutations as well as specific gene–gene interactions, and about possible treatment effects of midostaurin. Despite some limitations (e.g., the study was not powered for genetic subgroup analysis and results are of exploratory nature) our study provides the basis for future studies and validation of the findings in an independent dataset. Here, randomized trials comparing midostaurin with next-generation FLT3 inhibitors in patients with *FLT3* mutations (e.g., NCT04027309) are currently underway and likely will provide additional valuable data with regard to the underlying genetic landscape and its clinical implications.

## Supplementary information

Supplemental Appendix

Mutational Dataset

## Data Availability

A Supplementary Appendix accompanies this paper on the Leukemia website. Mutational data are available as.xlsx file as part of the Supplementary Information. Raw data were generated at the University of Ulm and are available on request from the corresponding author (KD).
