# Supplementary material for: Genomic landscape of patients with FLT3-mutated acute myeloid leukemia (AML) treated within the CALGB 10603/RATIFY trial
Source: Leukemia. 2022 Aug 3;36(9):2218–27. doi: 10.1038/s41375-022-01650-w (PMC9417991; doi:10.1038/s41375-022-01650-w)
Supplement: Supplementary file 1 — Supplemental Appendix [file 41375_2022_1650_MOESM1_ESM.pdf]

Supplemental Information

GENOMIC LANDSCAPE OF PATIENTS WITH *FLT3*-MUTATED ACUTE MYELOID LEUKEMIA (AML) TREATED WITHIN THE CALGB 10603/RATIFY TRIAL

AUTHORS

Nikolaus Jahn[1,*], Ekaterina Jahn[1,*], Maral Saadati[2], Lars Bullinger[3], Richard A. Larson[4], Tiziana Ottone[5,6], Sergio Amadori[5], Thomas W. Prior[7], Joseph M. Brandwein[8], Frederick R. Appelbaum[9], Bruno C. Medeiros[10], Martin S. Tallman[11], Gerhard Ehninger[12], Michael Heuser[13], Arnold Ganser[13], Celine Pallaud[14], Insa Gathmann[14], Julia Krzykalla[15], Axel Benner[15], Clara D. Bloomfield[16], Christian Thiede[12], Richard M. Stone[17], Hartmut Döhner[1], and Konstanze Döhner[1]

[1]Department of Internal Medicine III, University Hospital of Ulm, Ulm, Germany; [2]Saadati Solutions, Ladenburg, Germany; [3]Department of Hematology, Oncology and Tumor Immunology, Charité University, Berlin, Germany; [4]Department of Medicine and Comprehensive Cancer Center, University of Chicago, Chicago, IL, USA; [5]Department of Biomedicine and Prevention, University Tor Vergata, Rome, Italy; [6]Santa Lucia Foundation, I.R.C.C.S., Neuro-Oncohematology, Rome, Italy; [7]Case Western Reserve University, Cleveland, OH, USA; [8]Department of Medicine, University of Alberta, Edmonton, Canada; [9]Clinical Research Division, Fred Hutchinson Cancer Research Center, Seattle, WA, USA; [10]Division of Hematology, Stanford Comprehensive Cancer Center, Stanford University, Stanford, CA, USA; [11]Division of Hematologic Malignancies, Leukemia Service, Memorial Sloan Kettering Cancer Center, New York, NY; [12]Medizinische Klinik und Poliklinik I, Universitätsklinikum Carl Gustav Carus der TU Dresden, Dresden, Germany; [13]Department of Hematology, Hemostasis, Oncology and Stem Cell Transplantation, Hannover Medical School, Hannover, Germany; [14]Novartis Pharmaceuticals, Basel, Switzerland; [15]Division of Biostatistics, German Cancer Research Center Heidelberg, Heidelberg, Germany; [16]The Ohio State University Comprehensive Cancer Center, Columbus, OH, USA; [17]Department of Medical Oncology, Dana-Farber/Partners CancerCare, Boston, MA, USA

*N.J. and E.J. contributed equally to this work

SUPPLEMENTAL METHODS **3**
Library Enrichment and Sequencing 3
Variant calling 4
Curation of oncogenic variants 4
SUPPLEMENTAL TABLES **6**
SUPPLEMENTAL FIGURES **17**

SUPPLEMENTAL METHODS

Library Enrichment and Sequencing

The library enrichment was performed using the commercially available SureSelectXT in-solution capture technology from Agilent Technologies (Santa Clara, CA, USA). The library comprised the entire coding region of 262 candidate genes involved in hematological malignancies including 20 kinases targeted by midostaurin. For library design Agilent's online tool SureDesign was used. Only exons with a consensus annotation in the RefSeq, Ensembl, CCDS, Gencode, and SNP databases were considered relevant. The UCSC human genome 19 (H. sapiens, hg19, GRCh37, February 2009) served as reference genome for design and determination of genomic coordinates.

Genomic DNA (200ng per sample) extracted from pre-treatment bone marrow (409, 86%) or peripheral blood (66, 14%) specimens was used for molecular screening. SureSelect library preparation and indexing were performed following the manufacturer's instructions for Illumina paired-end sequencing. Samples were then transferred to a cBot (Illumina, San Diego, CA, USA) to create clonal clusters on a flow cell by bridge amplification (Illumina reagent kit: TruSeq PE Cluster Kit v3-cBot-HS). Finally, 2x 100 bp paired-end sequencing by synthesis was carried out on a HiSeq2000 (Illumina, San Diego, CA, USA) using Illumina's TruSeq SBS Kit v3-HS reagents.

Variant calling

The sequencing quality of each sample was assessed using the NGS QC toolkit (2.3.3) and, where necessary, adapter and read end trimming were performed using cutadapt (1.8.3) and in-house scripting respectively.

Paired-end reads were then aligned to the hg19 reference using BWA-MEM (0.7.10). Alignments are sorted and indexed by Picard (1.138) and locally realigned using GATK (3.4.46). For each sample, coverage statistics were calculated using BEDTools (2.24.0) and processed by SAMtools (0.1.19). VarScan2 (2.3.9) was then used for variant calling within the target regions sequenced. All variants were annotated by Annovar (release 22Mar2015) but only non-synonymous mutations affecting exons or splice sites were retained. These were further filtered to remove calls within known regions of segmental duplication, variants annotated in dbSNP (138) but not COSMIC (70) and variants with a minor allele frequency (MAF) above 0.01 in either the 1000 Genomes Project or the Exome Sequencing Project (ESP 6500).

Curation of oncogenic variants

All calls yielded by the computational annotation workflow were subject to further curation. Only variants considered oncogenic were included in the subsequent analyses. The algorithm for mutation reporting is as follows:

a) Removal of all variants that are annotated in SNP databases and occur with a minor allele frequency (MAF) >0.001 in the 1000 Genomes Project, dbSNP150 or the Exome Sequencing Project (ESP 6500).

b) Removal of variants present within regions prone to sequence context specific artifacts, including regions of high depth, enriched for reads of low mapping quality that harbor multiple mismatches

c) Removal of all one bp insertions or deletions present adjacent to regions of more than 5 homopolymer bases (for example insG adjacent to GGGGG) and a variant allele frequency of ≤0.1

d) Removal of all missense variants with a variant allele frequency between ≥0.45 and ≤0.55 or ≥0.9 and 1.0, indicative of polymorphisms, unless they are present with ≥5 counts in COSMIC database (v85) and with ≥1 confirmed somatic.

e) Retention of all frameshift, nonsense or splicing variants with a variant allele frequency ≥0.03

f) Retention of all missense variants with a variant allele frequency between 0.03 and <0.45 or >0.55 and <0.9, indicative of (likely) oncogenic variants.

SUPPLEMENTAL TABLES

Supplemental Table S1: Comparison of clinical characteristics of patients included and excluded into this analysis of entire CALGB 10603/RATIFY trial cohort (N=717).

Characteristic	Excluded, N = 242[1]	Included, N = 475[1]	p-value[2]
**Age at registration**	48 (40, 53)	48 (39, 54)	1
**WBC count at baseline (10E9/L)**	34 (13, 89)	35 (12, 72)	0.5
**Unknown**	7	3	
**Sex**			0.6
**Male**	111 (46%)	208 (44%)	
**Female**	131 (54%)	267 (56%)	
**ECOG performance category**			0.5
**0-1**	211 (87%)	422 (89%)	
**2**	31 (13%)	53 (11%)	
***FLT3* mutation type**			0.073
**TKD**	47 (20%)	116 (24%)	
**ITD allelic ratio <0.5**	82 (34%)	126 (27%)	
**ITD allelic ratio >=0.5**	109 (46%)	232 (49%)	
**Unknown**	4	1	
**Treatment**			0.022
**Midostaurin**	107 (44%)	253 (53%)	
**Placebo**	135 (56%)	222 (47%)	

[1] Median (IQR); n (%)

[2] Wilcoxon rank sum test; Pearson's Chi-squared test

Supplemental Table S2: List of all genes targeted by custom sequencing panel

ABCA12	*CDKN2B*	*DNMT3B*	*GNB1*	*MAP3K4*	*NXF1*	*RASGRF1*	*STAG1*
ABL1	*CDKN2C*	*DYNC1H1*	*H3F3A*	*MAP3K9*	*OBSCN*	*RB1*	*STAG2*
ACIN1	*CEBPA*	*EED*	*H3F3B*	*MGA*	*OMG*	*RBBP5*	*STAT3*
ACSS3	*CHEK2*	*EEFSEC*	*HAX1*	*MLL3/KMT2C*	*PAX5*	*RBBP6*	*STAT5A*
ADGRV1	*CLTCL1*	*EGFR*	*HCN1*	*MLL5/KMT2E*	*PDGFB*	*RBMX*	*SUZ12*
ALK	*CNNM2*	*ELANE*	*HIPK2*	*MN1*	*PDGFRB*	*RET*	*SYNE1*
ANKRD26	*COPRS*	*EP300*	*HNRNPK*	*MPL*	*PDPK1*	*RHOA*	*TCIRG1*
ARHGEF10	*CREBBP*	*EPHA6*	*HRAS*	*MST1*	*PHF6*	*RMI1*	*TERC*
ARID1A	*CSF1R*	*ETNK1*	*IDH1*	*MYC*	*PHIP*	*ROBO1*	*TERT*
ARID2	*CSF2RB*	*ETV6*	*IDH2*	*MYH9*	*PHKG1*	*ROBO2*	*TET1*
AS3MT	*CSF3R*	*EVI2A*	*IKZF2*	*MYLK2*	*PIK3CA*	*RPS6KA2*	*TET2*
ASXL1	*CSMD1*	*EVI2B*	*INPP5D*	*MYO1F*	*PKN2*	*RPS6KA3*	*TINF2*
ASXL2	*CSMD2*	*EWSR1*	*IRF1*	*NCOA7*	*PLEKHH1*	*RPS6KA6*	*TNK1*
ATRX	*CSNK1A1*	*EZH1*	*IRF4*	*NDE1*	*PLEKHS1*	*RRAS*	*TNK2*
BAP1	*CTC1*	*EZH2*	*IRF8*	*NEK2*	*PPM1D*	*RUNX1*	*TP53*

BCL10	*CTCF*	*FAM175A*	*JAK1*	*NF1*	*PRKAG2*	*RYR2*	*TTC39A*
BCL2	*CTNNB1*	*FAM5C*	*JAK2*	*NF2*	*PRKG2*	*SAMHD1*	*U2AF1*
BCOR	*CUX1*	*FAT4*	*JAK3*	*NFE2*	*PRPF40A*	*SETBP1*	*U2AF2*
BCORL1	*DCC*	*FBXW7*	*JARID2*	*NFE2L1*	*PRPF40B*	*SETD2*	*UBQLN1*
BCR	*DDX23*	*FGFR2*	*KAT6A*	*NFE2L2*	*PRPF8*	*SETDB1*	*UBXN11*
BRAF	*DDX4*	*FLG*	*KDM5C*	*NIPBL*	*PTEN*	*SF1*	*WAC*
BRCC3	*DDX41*	*FLT3*	*KDM6A*	*NOTCH1*	*PTPN11*	*SF3A1*	*WHSC1*
C6	*DDX54*	*FOXP1*	*KDR*	*NOTCH2*	*PTPRF*	*SF3B1*	*WRAP53*
C9orf103	*DHX15*	*FRMD3*	*KIF27*	*NPM1*	*PTPRT*	*SH2B3*	*WT1*
CALR	*DHX33*	*G6PC3*	*KIT*	*NRAS*	*PXDN*	*SMARCB1*	*YLPM1*
CBL	*DICER1*	*GALNT11*	*KMT2A*	*NRXN1*	*RAB11FIP4*	*SMC1A*	*ZBTB33*
CCDC26	*DIS3*	*GALNTL5*	*KMT2D*	*NRXN3*	*RAC1*	*SMC3*	*ZBTB7A*
CCND1	*DKC1*	*GATA1*	*KRAS*	*NSD1*	*RAD21*	*SMG1*	*ZMYM3*
CCND2	*DNAH9*	*GATA2*	*LAMA1*	*NT5C2*	*RAD50*	*SPI1*	*ZNF318*
CDHR1	*DNAJB8*	*GFI1*	*LAMC3*	*NTRK1*	*RAD51*	*SPRED2*	*ZNF687*
CDK4	*DND1*	*GIGYF2*	*LUC7L2*	*NTRK3*	*RASA2*	*SRCAP*	*ZRSR2*
CDKN1B	*DNM2*	*GKAP1*	*MAP3K10*	*NUMA1*	*RASA3*	*SRP72*	
CDKN2A	*DNMT3A*	*GNAS*	*MAP3K11*	*NUP98*	*RASEF*	*SRSF2*	

Supplemental Table S3: Genes targeted by midostaurin (Midostaurin kinome)

JAK3	*KDR*	*KIT*	*MAP3K10*	*MAP3K11*	*MAP3K9*	*MST1*	*NTRK1*	*NTRK3*	*PDGFRB*
PDPK1	*PHKG1*	*PKN2*	*PRKG2*	*RET*	*RPS6KA2*	*RPS6KA3*	*RPS6KA6*	*TNK1*	*TNK2*

Supplemental Table S4: Frequency of gene mutations overall and by *FLT3* mutational subgroups

**Characteristic**	**Overall, N = 475**[1]	**TKD, N = 116**[1]	**ITD, N = 359**[1]	**p**[2]
NPM1	291 (61%)	71 (61%)	220 (61%)	1.00
DNMT3A	187 (39%)	44 (38%)	143 (40%)	0.74
WT1	100 (21%)	16 (14%)	84 (23%)	0.03
TET2	55 (12%)	10 (8.6%)	45 (13%)	0.32
RUNX1	53 (11%)	10 (8.6%)	43 (12%)	0.40
NRAS	53 (11%)	28 (24%)	25 (7.0%)	<0.001
PTPN11	45 (9.5%)	16 (14%)	29 (8.1%)	0.10
IDH1	39 (8.2%)	9 (7.8%)	30 (8.4%)	1.00
ASXL1	38 (8.0%)	5 (4.3%)	33 (9.2%)	0.12
IDH2 (R140)	34 (7.2%)	7 (6.0%)	27 (7.5%)	0.68
SMC1A	28 (5.9%)	12 (10%)	16 (4.5%)	0.04
CEBPA	26 (5.5%)	2 (1.7%)	24 (6.7%)	0.06
SMC3	24 (5.1%)	9 (7.8%)	15 (4.2%)	0.14
RAD21	23 (4.8%)	8 (6.9%)	15 (4.2%)	0.22
BCOR	20 (4.2%)	3 (2.6%)	17 (4.7%)	0.43
KMT2D	18 (3.8%)	1 (0.9%)	17 (4.7%)	0.09
STAG2	18 (3.8%)	6 (5.2%)	12 (3.3%)	0.40
BCORL1	16 (3.4%)	5 (4.3%)	11 (3.1%)	0.56

ZBTB7A	15 (3.2%)	6 (5.2%)	9 (2.5%)	0.22
MYC	15 (3.2%)	5 (4.3%)	10 (2.8%)	0.38
GATA2	14 (2.9%)	2 (1.7%)	12 (3.3%)	0.53
OBSCN	14 (2.9%)	3 (2.6%)	11 (3.1%)	1.00
KRAS	13 (2.7%)	6 (5.2%)	7 (1.9%)	0.10
NFE2	13 (2.7%)	1 (0.9%)	12 (3.3%)	0.20
CSMD1	11 (2.3%)	3 (2.6%)	8 (2.2%)	0.73
KDM6A	11 (2.3%)	4 (3.4%)	7 (1.9%)	0.48
MGA	11 (2.3%)	3 (2.6%)	8 (2.2%)	0.73
SETD2	10 (2.1%)	2 (1.7%)	8 (2.2%)	1.00
SYNE1	10 (2.1%)	1 (0.9%)	9 (2.5%)	0.46
CREBBP	10 (2.1%)	4 (3.4%)	6 (1.7%)	0.27
ARID2	9 (1.9%)	4 (3.4%)	5 (1.4%)	0.23
MN1	9 (1.9%)	4 (3.4%)	5 (1.4%)	0.23
SF3B1	9 (1.9%)	2 (1.7%)	7 (1.9%)	1.00
SRSF2	8 (1.7%)	3 (2.6%)	5 (1.4%)	0.41
KIT	8 (1.7%)	5 (4.3%)	3 (0.8%)	0.02
ZBTB33	7 (1.5%)	2 (1.7%)	5 (1.4%)	0.68
HNRNPK	7 (1.5%)	3 (2.6%)	4 (1.1%)	0.37
NF1	7 (1.5%)	1 (0.9%)	6 (1.7%)	1.00
PTPRF	6 (1.3%)	0 (0%)	6 (1.7%)	0.34
ZNF318	6 (1.3%)	1 (0.9%)	5 (1.4%)	1.00
NOTCH1	6 (1.3%)	2 (1.7%)	4 (1.1%)	0.64
PHF6	6 (1.3%)	0 (0%)	6 (1.7%)	0.34
CSMD2	6 (1.3%)	4 (3.4%)	2 (0.6%)	0.03
CBL	6 (1.3%)	0 (0%)	6 (1.7%)	0.34
DNAH9	6 (1.3%)	2 (1.7%)	4 (1.1%)	0.64
GNAS	6 (1.3%)	1 (0.9%)	5 (1.4%)	1.00
KMT2A	6 (1.3%)	1 (0.9%)	5 (1.4%)	1.00
FOXP1	6 (1.3%)	4 (3.4%)	2 (0.6%)	0.03
CSF3R	6 (1.3%)	1 (0.9%)	5 (1.4%)	1.00
EP300	6 (1.3%)	1 (0.9%)	5 (1.4%)	1.00
ACIN1	5 (1.1%)	0 (0%)	5 (1.4%)	0.34
RYR2	5 (1.1%)	0 (0%)	5 (1.4%)	0.34
ARID1A	5 (1.1%)	0 (0%)	5 (1.4%)	0.34
ASXL2	5 (1.1%)	1 (0.9%)	4 (1.1%)	1.00
LAMC3	5 (1.1%)	2 (1.7%)	3 (0.8%)	0.60
UBXN11	5 (1.1%)	0 (0%)	5 (1.4%)	0.34
ROBO1	5 (1.1%)	1 (0.9%)	4 (1.1%)	1.00
GPR98	5 (1.1%)	1 (0.9%)	4 (1.1%)	1.00
SRCAP	5 (1.1%)	2 (1.7%)	3 (0.8%)	0.60
EZH2	5 (1.1%)	1 (0.9%)	4 (1.1%)	1.00
FBXW7	5 (1.1%)	2 (1.7%)	3 (0.8%)	0.60
BCR	4 (0.8%)	1 (0.9%)	3 (0.8%)	
MYH9	4 (0.8%)	2 (1.7%)	2 (0.6%)	
ETV6	4 (0.8%)	1 (0.9%)	3 (0.8%)	
DNMT3B	4 (0.8%)	1 (0.9%)	3 (0.8%)	
LAMA1	4 (0.8%)	2 (1.7%)	2 (0.6%)	
U2AF1	4 (0.8%)	1 (0.9%)	3 (0.8%)	
NTRK3	4 (0.8%)	0 (0%)	4 (1.1%)	
CTCF	4 (0.8%)	0 (0%)	4 (1.1%)	
MAP3K11	4 (0.8%)	1 (0.9%)	3 (0.8%)	

CNNM2	4 (0.8%)	0 (0%)	4 (1.1%)
NSD1	4 (0.8%)	2 (1.7%)	2 (0.6%)
SH2B3	4 (0.8%)	0 (0%)	4 (1.1%)
KMT2E	4 (0.8%)	1 (0.9%)	3 (0.8%)
KDM5C	4 (0.8%)	2 (1.7%)	2 (0.6%)
BRCC3	4 (0.8%)	0 (0%)	4 (1.1%)
KMT2C	4 (0.8%)	1 (0.9%)	3 (0.8%)
CUX1	4 (0.8%)	3 (2.6%)	1 (0.3%)
TERT	4 (0.8%)	0 (0%)	4 (1.1%)
EPHA6	4 (0.8%)	1 (0.9%)	3 (0.8%)
FAT4	4 (0.8%)	0 (0%)	4 (1.1%)
INPP5D	4 (0.8%)	1 (0.9%)	3 (0.8%)
TP53	4 (0.8%)	2 (1.7%)	2 (0.6%)
ROBO2	3 (0.6%)	1 (0.9%)	2 (0.6%)
SETBP1	3 (0.6%)	1 (0.9%)	2 (0.6%)
CDHR1	3 (0.6%)	0 (0%)	3 (0.8%)
SF3A1	3 (0.6%)	1 (0.9%)	2 (0.6%)
PLEKHH1	3 (0.6%)	0 (0%)	3 (0.8%)
SETDB1	3 (0.6%)	0 (0%)	3 (0.8%)
EGFR	3 (0.6%)	2 (1.7%)	1 (0.3%)
RBBP6	3 (0.6%)	2 (1.7%)	1 (0.3%)
ATRX	3 (0.6%)	1 (0.9%)	2 (0.6%)
PDGFRB	3 (0.6%)	1 (0.9%)	2 (0.6%)
RPS6KA6	3 (0.6%)	2 (1.7%)	1 (0.3%)
NTRK1	3 (0.6%)	0 (0%)	3 (0.8%)
YLPM1	3 (0.6%)	0 (0%)	3 (0.8%)
RRAS	3 (0.6%)	1 (0.9%)	2 (0.6%)
KAT6A	3 (0.6%)	3 (2.6%)	0 (0%)
PPM1D	3 (0.6%)	2 (1.7%)	1 (0.3%)
HIPK2	3 (0.6%)	0 (0%)	3 (0.8%)
MAP3K10	2 (0.4%)	0 (0%)	2 (0.6%)
TCIRG1	2 (0.4%)	0 (0%)	2 (0.6%)
MPL	2 (0.4%)	1 (0.9%)	1 (0.3%)
DDX41	2 (0.4%)	0 (0%)	2 (0.6%)
RAD50	2 (0.4%)	0 (0%)	2 (0.6%)
WRAP53	2 (0.4%)	0 (0%)	2 (0.6%)
CCND2	2 (0.4%)	1 (0.9%)	1 (0.3%)
CSF1R	2 (0.4%)	0 (0%)	2 (0.6%)
CSF2RB	2 (0.4%)	1 (0.9%)	1 (0.3%)
DHX33	2 (0.4%)	0 (0%)	2 (0.6%)
WHSC1	2 (0.4%)	1 (0.9%)	1 (0.3%)
RPS6KA2	2 (0.4%)	1 (0.9%)	1 (0.3%)
MAP3K9	2 (0.4%)	1 (0.9%)	1 (0.3%)
FLG	2 (0.4%)	2 (1.7%)	0 (0%)
ABCA12	2 (0.4%)	0 (0%)	2 (0.6%)
CTC1	2 (0.4%)	0 (0%)	2 (0.6%)
HCN1	2 (0.4%)	1 (0.9%)	1 (0.3%)
JARID2	2 (0.4%)	0 (0%)	2 (0.6%)
DNM2	2 (0.4%)	0 (0%)	2 (0.6%)
SF1	2 (0.4%)	0 (0%)	2 (0.6%)
EED	2 (0.4%)	1 (0.9%)	1 (0.3%)
PDGFB	2 (0.4%)	2 (1.7%)	0 (0%)

RASA2	2 (0.4%)	0 (0%)	2 (0.6%)
GATA1	2 (0.4%)	0 (0%)	2 (0.6%)
TNK2	2 (0.4%)	0 (0%)	2 (0.6%)
MAP3K4	2 (0.4%)	0 (0%)	2 (0.6%)
JAK3	2 (0.4%)	1 (0.9%)	1 (0.3%)
PRPF40B	2 (0.4%)	1 (0.9%)	1 (0.3%)
DCC	2 (0.4%)	0 (0%)	2 (0.6%)
MYO1F	2 (0.4%)	1 (0.9%)	1 (0.3%)
DIS3	2 (0.4%)	0 (0%)	2 (0.6%)
TINF2	2 (0.4%)	0 (0%)	2 (0.6%)
ZMYM3	2 (0.4%)	0 (0%)	2 (0.6%)
ALK	2 (0.4%)	1 (0.9%)	1 (0.3%)
NUP98	2 (0.4%)	1 (0.9%)	1 (0.3%)
RMI1	2 (0.4%)	0 (0%)	2 (0.6%)
GFI1	2 (0.4%)	0 (0%)	2 (0.6%)
DYNC1H1	1 (0.2%)	1 (0.9%)	0 (0%)
PTPRT	1 (0.2%)	0 (0%)	1 (0.3%)
RET	1 (0.2%)	0 (0%)	1 (0.3%)
DDX54	1 (0.2%)	1 (0.9%)	0 (0%)
ACSS3	1 (0.2%)	0 (0%)	1 (0.3%)
EVI2B	1 (0.2%)	1 (0.9%)	0 (0%)
JAK1	1 (0.2%)	0 (0%)	1 (0.3%)
KDR	1 (0.2%)	0 (0%)	1 (0.3%)
DHX15	1 (0.2%)	0 (0%)	1 (0.3%)
STAT5A	1 (0.2%)	1 (0.9%)	0 (0%)
BRINP3	1 (0.2%)	1 (0.9%)	0 (0%)
BRAF	1 (0.2%)	1 (0.9%)	0 (0%)
IRF4	1 (0.2%)	1 (0.9%)	0 (0%)
COPRS	1 (0.2%)	0 (0%)	1 (0.3%)
PAX5	1 (0.2%)	0 (0%)	1 (0.3%)
PHIP	1 (0.2%)	0 (0%)	1 (0.3%)
RPS6KA3	1 (0.2%)	0 (0%)	1 (0.3%)
SMG1	1 (0.2%)	0 (0%)	1 (0.3%)
EEFSEC	1 (0.2%)	1 (0.9%)	0 (0%)
C6	1 (0.2%)	0 (0%)	1 (0.3%)
NFE2L1	1 (0.2%)	0 (0%)	1 (0.3%)
RASEF	1 (0.2%)	0 (0%)	1 (0.3%)
RASA3	1 (0.2%)	0 (0%)	1 (0.3%)
HRAS	1 (0.2%)	0 (0%)	1 (0.3%)
MYLK2	1 (0.2%)	0 (0%)	1 (0.3%)
RAB11FIP4	1 (0.2%)	0 (0%)	1 (0.3%)
DICER1	1 (0.2%)	1 (0.9%)	0 (0%)
TET1	1 (0.2%)	0 (0%)	1 (0.3%)
SPRED2	1 (0.2%)	0 (0%)	1 (0.3%)
GALNT11	1 (0.2%)	0 (0%)	1 (0.3%)
ZRSR2	1 (0.2%)	0 (0%)	1 (0.3%)
PRKAG2	1 (0.2%)	0 (0%)	1 (0.3%)
JAK2	1 (0.2%)	0 (0%)	1 (0.3%)
BAP1	1 (0.2%)	1 (0.9%)	0 (0%)
NF2	1 (0.2%)	0 (0%)	1 (0.3%)
RASGRF1	1 (0.2%)	0 (0%)	1 (0.3%)
TNK1	1 (0.2%)	1 (0.9%)	0 (0%)

IDNK	1 (0.2%)	0 (0%)	1 (0.3%)
INSRR.NTRK1	1 (0.2%)	1 (0.9%)	0 (0%)
NRXN1	1 (0.2%)	0 (0%)	1 (0.3%)
NXF1	1 (0.2%)	0 (0%)	1 (0.3%)
ANKRD26	1 (0.2%)	0 (0%)	1 (0.3%)
BCL10	1 (0.2%)	0 (0%)	1 (0.3%)
PRPF40A	1 (0.2%)	0 (0%)	1 (0.3%)
SUZ12	1 (0.2%)	0 (0%)	1 (0.3%)
PTEN	1 (0.2%)	0 (0%)	1 (0.3%)
RAD51	1 (0.2%)	0 (0%)	1 (0.3%)
PRPF8	1 (0.2%)	1 (0.9%)	0 (0%)
PLEKHS1	1 (0.2%)	1 (0.9%)	0 (0%)
SPI1	1 (0.2%)	1 (0.9%)	0 (0%)
GALNTL5	1 (0.2%)	1 (0.9%)	0 (0%)
SAMHD1	1 (0.2%)	0 (0%)	1 (0.3%)
NRXN3	1 (0.2%)	1 (0.9%)	0 (0%)
HAX1	1 (0.2%)	0 (0%)	1 (0.3%)
TTC39A	1 (0.2%)	0 (0%)	1 (0.3%)
DKC1	1 (0.2%)	0 (0%)	1 (0.3%)
PHKG1	1 (0.2%)	1 (0.9%)	0 (0%)
WAC	1 (0.2%)	0 (0%)	1 (0.3%)
NUMA1	1 (0.2%)	0 (0%)	1 (0.3%)
FGFR2	1 (0.2%)	0 (0%)	1 (0.3%)
GIGYF2	1 (0.2%)	0 (0%)	1 (0.3%)
RBBP5	1 (0.2%)	0 (0%)	1 (0.3%)
NCOA7	1 (0.2%)	0 (0%)	1 (0.3%)

[1] *n (%)*
[2] *Fisher's exact test (p-value not adjusted for multiple testing)*

Supplemental Table S5: Functional categorization of recurrently mutated genes (>1%).

Methylation	Chromatin	Cohesin	Splicing	Signaling	Transcription	Other	Tumor suppressor
DNMT3A	*ACIN1*	*RAD21*	*HNRNPK*	*CBL*	*ARID1A*	*CSMD2*	*CSMD1*
IDH1	*ARID2*	*SMC1A*	*SF1*	*CSF3R*	*CEBPA*	*DNAH9*	*TP53*
IDH2	*ASXL1*	*SMC3*	*SF3A1*	*FLT3*	*ETV6*	*FBXW7*	
TET2	*ASXL2*	*STAG2*	*SF3B1*	*GNAS*	*FOXP1*	*LAMC3*	
	BCOR		*SRSF2*	*GPR98*	*GATA2*	*NPM1*	
	BCORL1		*U2AF1*	*KIT*	*MGA*	*OBSCN*	
	CREBBP		*ZRSR2*	*KRAS*	*MN1*	*ROBO1*	
	EP300			*NF1*	*MYC*	*RYR2*	
	EZH2			*NOTCH1*	*NFE2*	*SYNE1*	
	KDM6A			*NRAS*	*PHF6*		
	KMT2A			*PTPN11*	*RUNX1*		
	KMT2D			*PTPRF*	*WT1*		
	KMT2E			*UBXN11*	*ZBTB33*		
	KMT2C				*ZBTB7A*		
	SETD2				*ZNF318*		
	SRCAP						

Supplemental Table S6: Genomic classes according to Papaemmanuil E, Gerstung M et al NEJM 2016.

**Genomic subgroup (long)**	**Genomic subgroup (short)**
AML with inv(16)(p13.1q22) or t(16;16)(p13.1;q22); CBFB–MYH11	inv(16)
AML with t(8;21)(q22;q22); RUNX1–RUNX1T1	t(8;21)
AML with MLL fusion genes; t(x;11)(x;q23)	t(11q23;x)
AML with inv(3)(q21q26.2) or t(3;3)(q21;q26.2); GATA2, MECOM(EVI1)	inv(3)
AML with t(6;9)(p23;q34); DEK–NUP214	t(6;9)
AML with t(15;17)(q22;q12); PML–RARA	t(15;17)
AML with NPM1 mutation	NPM1
AML with biallelic CEBPA mutations*	$CEBPA^{biallelic}$
AML with TP53 mutations, chromosomal aneuploidy, or both#	TP53-aneuploidy
AML with mutated chromatin, RNA-splicing genes, or both+	Chromatin-Spliceosome
AML with IDH2R172 mutations and no other class-defining lesions	$IDH2^{R172}$
AML meeting criteria for ≥2 genomic subgroups	2 classes
AML with driver mutations but no detected class-defining lesions	No class
AML with no detected driver mutations	No drivers detected

* Patients with two different mutations in *CEBPA*

# Classification in this subgroup requires *TP53* mutation, complex karyotype [3 or more abnormalities, in the absence of 1 of the WHO-designated recurring translocations or inversions, that is, t(8;21), inv(16) or t(16;16), t(9;11),t(v;11)(v;q23.3), t(6;9), inv(3) or t(3;3); AML with t(9;22)], or in the absence of other class-defining lesions, one or more of the following: −7/7q, −5/5q, −4/4q, −9q, −12/12p, −17/−17p, −18/18q, −20/20q, +11/11q, +13, +21, or +22.

+ Classification in this subgroup requires one or more driver mutations in *RUNX1*, *ASXL1*, *BCOR*, *STAG2*, *EZH2*, *SRSF2*, *SF3B1*, *U2AF1*, *ZRSR2*, or *MLL*-PTD. In the presence of other class-defining lesions — namely, inv(16), t(15;17), t(8;21), t(6;9), inv(3), MLL fusion genes, or complex karyotype or driver mutations in *TP53*, *NPM1*, or $CEBPA^{biallelic}$ — two or more chromatin–spliceosome mutations are required.

Supplemental Table S7: Baseline characteristics of genomic AML classes in the cohort of 451 of 475 patients, in which subcategorization into genomic AML classes was possible.

	NPM1 **(N=287)**	**CBF (N=18)**	***TP53-aneuploidy*** **(N=18)**	**Chromatin-spliceosome (N=68)**	**No Class (N=60)**	**Overall (N=451)**
**Age** (years)						
Mean (SD)	46.9 (10.1)	44.3 (10.4)	46.3 (9.80)	47.2 (10.5)	39.6 (12.2)	45.9 (10.7)
Median [Min,Max]	48.8 [18.0,59.9]	44.3 [23.4,57.5]	48.4 [22.0,57.4]	50.2 [19.5,59.8]	38.8 [19.4,59.1]	48.1 [18.0,59.9]
**Sex**						
Male	109 (38.0%)	10 (55.6%)	7 (38.9%)	36 (52.9%)	31 (51.7%)	193 (42.8%)
Female	178 (62.0%)	8 (44.4%)	11 (61.1%)	32 (47.1%)	29 (48.3%)	258 (57.2%)
FLT3 **mutation type**						
TKD	70 (24.4%)	12 (66.7%)	7 (38.9%)	12 (17.6%)	8 (13.3%)	109 (24.2%)
ITD <0.5 allelic ratio	74 (25.8%)	3 (16.7%)	5 (27.8%)	21 (30.9%)	16 (26.7%)	119 (26.4%)
ITD ≥0.5 allelic ratio	143 (49.8%)	3 (16.7%)	6 (33.3%)	35 (51.5%)	35 (58.3%)	222 (49.2%)
Missing	0 (0%)	0 (0%)	0 (0%)	0 (0%)	1 (1.7%)	1 (0.2%)
**WBC count at baseline (10E9/L)**						
Mean (SD)	54.8 (55.8)	41.6 (43.7)	40.9 (45.3)	43.8 (47.3)	47.1 (45.7)	51.1 (52.5)
Median [Min,Max]	39.6 [1.40,330]	26.0 [1.40,159]	25.9 [2.70,154]	28.6 [0.600,236]	30.2 [1.20,207]	35.5 [0.600,330]
Missing	1 (0.3%)	0 (0%)	1 (5.6%)	1 (1.5%)	0 (0%)	3 (0.7%)
**ECOG performance status**						
0-1	255 (88.9%)	17 (94.4%)	17 (94.4%)	64 (94.1%)	50 (83.3%)	403 (89.4%)
2	32 (11.1%)	1 (5.6%)	1 (5.6%)	4 (5.9%)	10 (16.7%)	48 (10.6%)
**Treatment**						
Placebo	136 (47.4%)	9 (50.0%)	8 (44.4%)	31 (45.6%)	26 (43.3%)	210 (46.6%)
Midostaurin	151 (52.6%)	9 (50.0%)	10 (55.6%)	37 (54.4%)	34 (56.7%)	241 (53.4%)
**Allogeneic HCT in CR1**						
No	211 (73.5%)	13 (72.2%)	16 (88.9%)	48 (70.6%)	44 (73.3%)	332 (73.6%)
Yes	76 (26.5%)	5 (27.8%)	2 (11.1%)	20 (29.4%)	16 (26.7%)	119 (26.4%)

Supplemental Table S8: Impact of 12 most frequent gene mutations on overall and event-free survival. Log rank test p-values from the univariate tests are indicated without (raw) and with adjustment (adj) for multiple testing via the Bonferroni-Holm procedure (FDR). See corresponding Kaplan Meier estimates in Supplemental Figure 2 and 3). Abbreviations: CI, confidence interval; HR, hazard ratio.

	Overall Surival					Event-free Survival				
	**Logrank p-value (raw)**	**Logrank p-value (adj)**	**HR**	**Lower CI**	**Upper CI**	**Logrank p-value (raw)**	**Logrank p-value (adj)**	**HR**	**Lower CI**	**Upper CI**
NPM1	<0.001	<0.001	0.60	0.46	0.77	<0.001	<0.001	0.60	0.48	0.74
WT1	<0.001	<0.001	1.83	1.38	2.43	0.018	0.195	1.36	1.05	1.75
ASXL1	0.013	0.125	1.67	1.11	2.52	0.100	0.903	0.72	0.48	1.07
IDH2	0.130	1	0.65	0.37	1.14	0.075	0.748	0.64	0.38	1.05
NRAS	0.097	0.875	0.68	0.43	1.08	0.793	1	1.05	0.71	1.55
DNMT3A	0.984	1	1.00	0.77	1.30	0.275	1	0.88	0.71	1.10
IDH1	0.773	1	1.07	0.68	1.69	0.649	1	0.91	0.61	1.35
PTPN11	0.287	1	0.77	0.48	1.25	0.204	1	0.75	0.48	1.17
RUNX1	0.141	1	1.32	0.91	1.90	0.310	1	0.83	0.58	1.19
SMC1A	0.219	1	0.69	0.37	1.26	0.183	1	1.25	0.90	1.73
SMC3	0.841	1	0.94	0.50	1.77	0.433	1	0.81	0.47	1.38
TET2	0.460	1	0.85	0.56	1.30	0.600	1	1.09	0.79	1.52

Supplemental Table S9: 4-year overall survival rates by genomic AML classes in the cohort of 451 of 475 patients, in which subcategorization into genomic AML classes was possible.

	**N**	**Number at risk**	**Events**	**Survival**	**Lower 95% CI**	**Higher 95% CI**
NPM1	287	136	120	56.9%	0.51	0.63
**CBF**	18	11	5	72.2%	0.54	0.96
***TP53*-aneuploidy**	18	5	11	35.3%	0.19	0.67
**Chromatin-spliceosome**	68	18	45	32.9%	0.23	0.46
**No class**	60	16	36	36.5%	0.26	0.52

Supplemental Table S10: 4-year event-free survival rates by genomic AML classes in the cohort of 451 of 475 patients, in which subcategorization into genomic AML classes was possible.

	N	Number at risk	Events	Survival	Lower 95% CI	Higher 95% CI
NPM1	287	87	177	37.7%	0.32	0.44
**CBF**	18	7	9	50.0%	0.32	0.79
***TP53*-aneuploidy**	18	1	17	5.6%	0.01	0.37
**Chromatin-spliceosome**	68	8	56	16.3%	0.09	0.28
**No class**	60	3	53	9.5%	0.04	0.22

Supplemental Table S11: Cox proportional hazard model for predictive impact of *FLT3* mutation type on hazard of death or event after treatment with midostaurin in cohort of 451 of 475 patients, in which subcategorization into genomic AML classes was possible. A hazard ratio of >1 indicates a higher and a hazard ratio of <1 a lower risk of death, respectively. Abbreviations: CBF, Core-binding factor AML; CI.95, 95% confidence interval; CR1, first complete remission; ECOG, Eastern Cooperative Oncology Group; HR, hazard ratio; HCT, hematopoietic cell transplantation; WBC, white blood cell count.

	**Overall survival**		**Event-free survival**	
**Variable**	HR [CI.95]	p-value	HR [CI.95]	p-value
**Genomic classes**				
CBF vs *NPM1*	0.67 [0.27;1.67]	0.392	0.80 [0.40;1.58]	0.522
TP53-aneuploidy vs *NPM1*	1.94 [1.04;3.61]	0.037	3.67 [2.21;6.09]	<0.001
Chromatin-Spliceosome vs *NPM1*	2.27 [1.61;3.21]	<0.001	1.89 [1.38;2.57]	<0.001
No Class vs *NPM1*	2.13 [1.43;3.17]	<0.001	2.51 [1.79;3.50]	<0.001
**Age**	1.01 [0.99;1.02]	0.338	1.00 [0.99;1.01]	0.849
**Sex**				
Female vs male	1.00 [0.76;1.32]	0.995	1.33 [1.06;1.68]	0.015
**ECOG (0-1 vs 2)**	1.28 [0.85;1.92]	0.236	0.87 [0.60;1.27]	0.475
**log2WBC**	1.08 [1.00;1.17]	0.045	1.09 [1.02;1.16]	0.014
**Allogeneic HCT in CR1**	0.58 [0.40;0.83]	0.003	0.72 [0.49;1.06]	0.093
***FLT3* TKD**				
Midostaurin vs Placebo	0.59 [0.32;1.11]	0.100	0.70 [0.43;1.17]	0.174
***FLT3* ITD <0.5 allelic ratio**				
Midostaurin vs Placebo	0.56 [0.32;1.00]	0.049	0.61 [0.39;0.97]	0.035
***FLT3* ITD ≥0.5 allelic ratio**				
Midostaurin vs Placebo	0.66 [0.47;0.94]	0.019	0.67 [0.49;0.91]	0.009
**Placebo**				
ITD <0.5 allelic ratio vs TKD	1.13 [0.64;2.01]	0.665	1.40 [0.87;2.25]	0.163
ITD ≥0.5 allelic ratio vs TKD	1.49 [0.90;2.47]	0.126	1.52 [1.00;2.32]	0.052

**Midostaurin**				
ITD <0.5 allelic ratio vs TKD	1.08 [0.58;2.03]	0.811	1.22 [0.74;2.00]	0.430
ITD ≥0.5 allelic ratio vs TKD	1.65 [0.98;2.78]	0.058	1.44 [0.94;2.20]	0.090

SUPPLEMENTAL FIGURES

Supplemental Figure S1: Mutational exclusivity and co-occurrence of recurrently (>5% of cases) mutated genes. A) Gene pairs that co-occurred more frequently are indicated by blue colors, gene pairs that co-occurred seldom by orange colors. The top 12 genes were tested for mutual exclusivity (66 possible combinations) resulting in 21 significant pairs before adjustment for multiple testing and 5 significant pairs after FDR adjustment: *NPM1-RUNX1* (p<.001), *DNMT3A-WT1* (p<.001), *DNMT3A-RUNX1* (p=.003), *NPM1-WT1* (p=.008) and *IDH2-TET2* (p=.021). Similarly, all combinations were tested for co-occurrence resulting in 2 significant pairs before adjustment for multiple testing: *NPM1-DNMT3A* and *IDH1-PTPN11*, which were not significant after FDR adjustment B) Width of bands reflects the number of cases in which mutations of corresponding genes co-occurred.

A

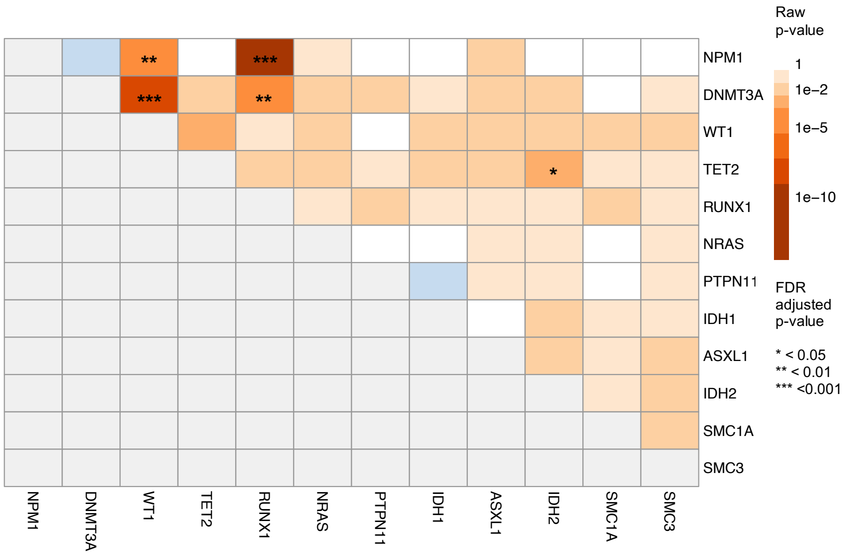

B

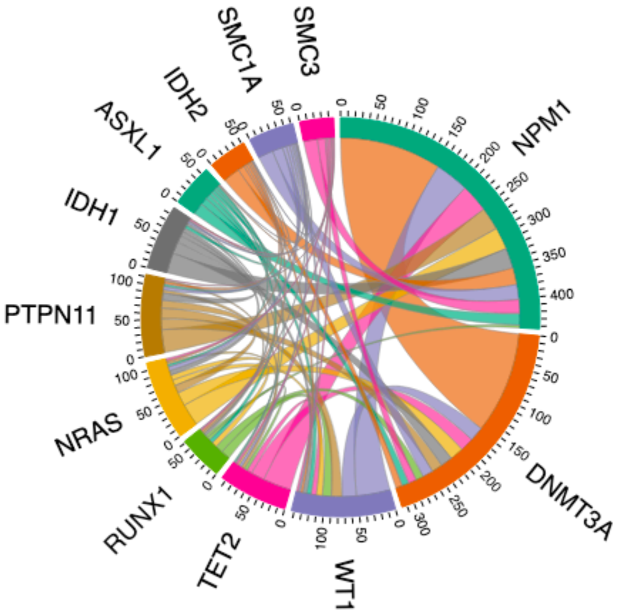

Supplemental Figure S2: Impact of gene mutations on overall survival. Log rank test p-values from the univariate tests are indicated without adjustment for multiple testing. Adjusted p-values are given in Supplemental Table S8.

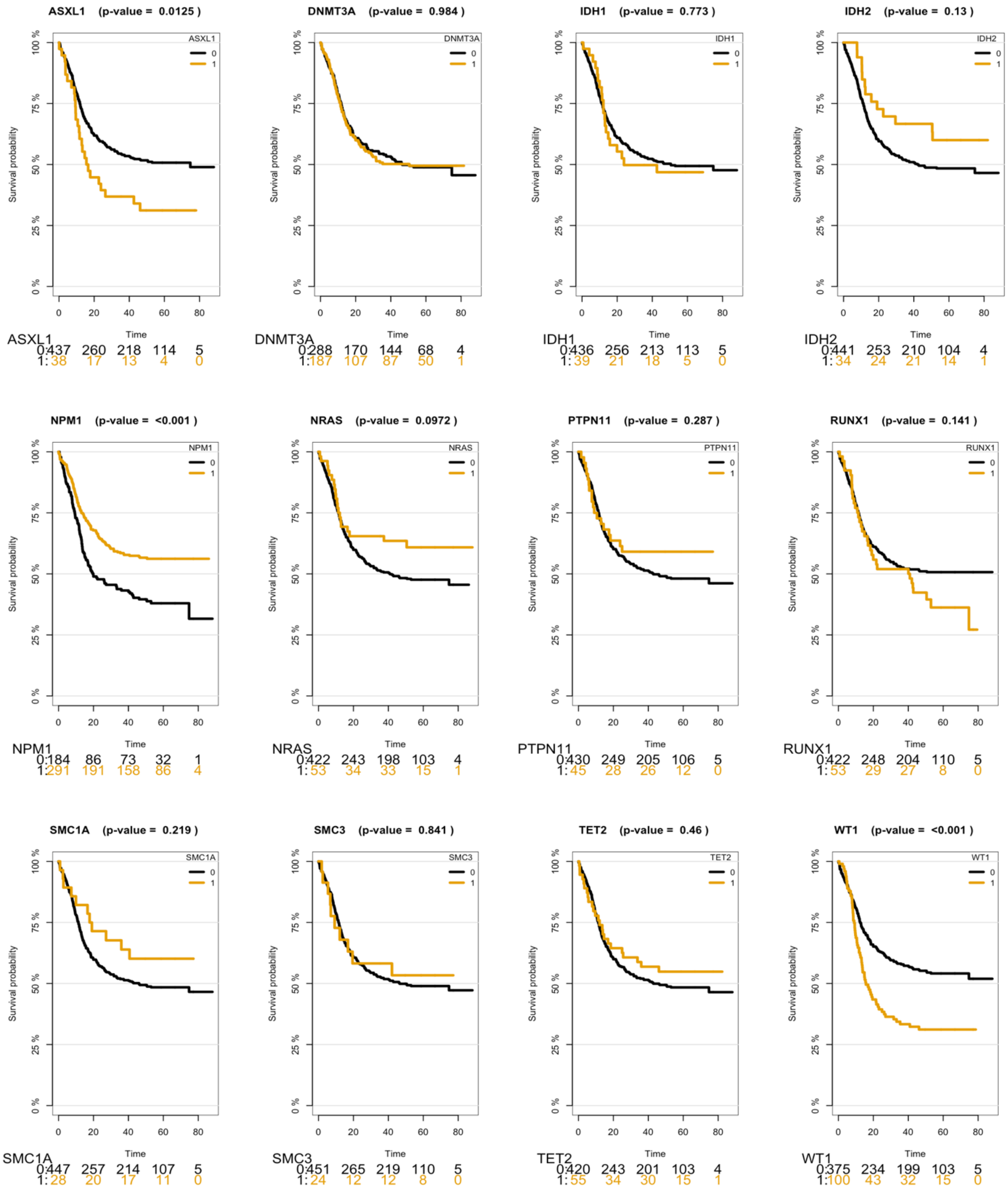

Supplemental Figure S3: Impact of gene mutations on event-free survival. Log rank test p-values from the univariate tests are indicated without adjustment for multiple testing. Adjusted p-values are given in Supplemental Table S8.

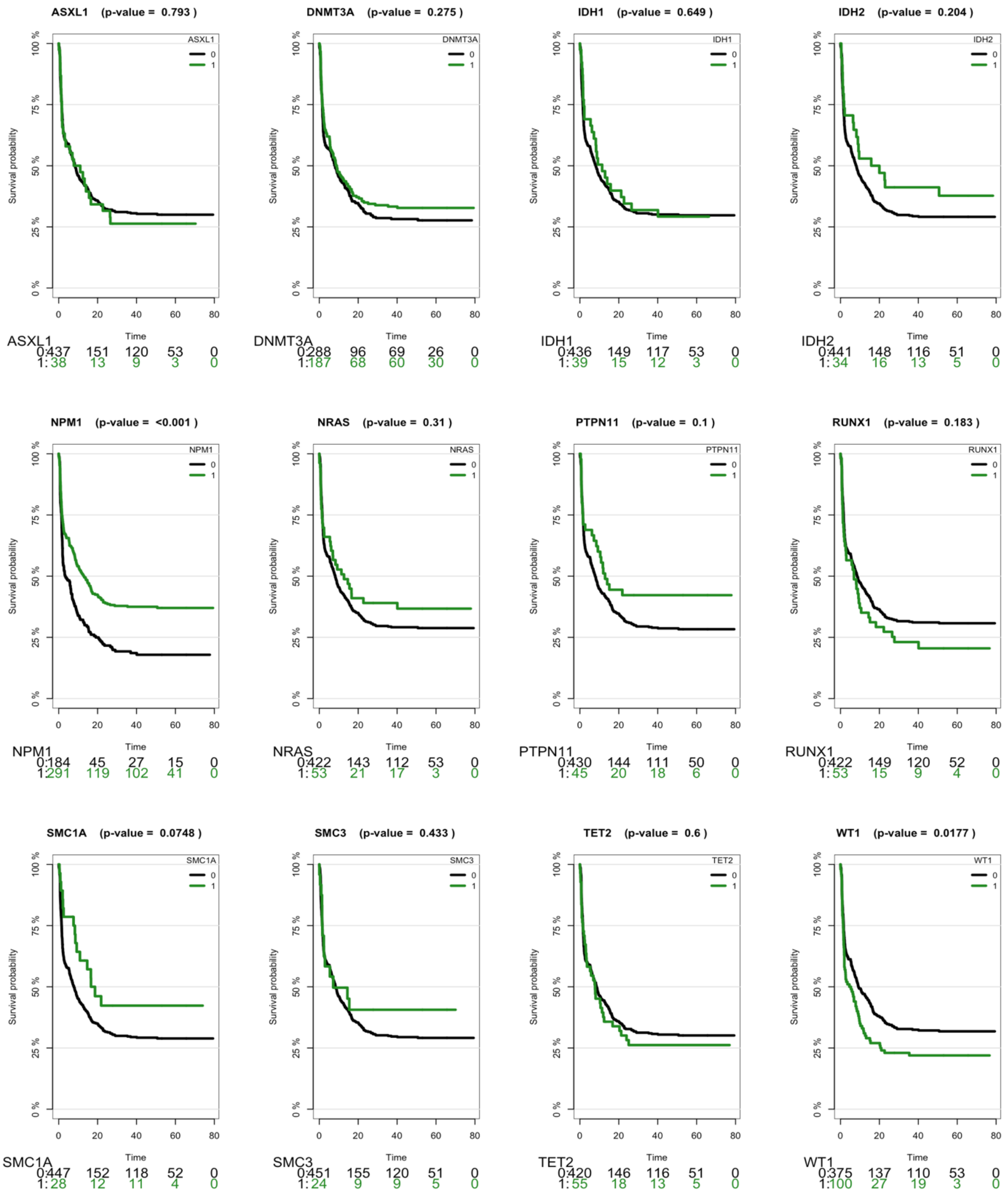

Supplemental Figure S4: Impact of *WT1* mutations on overall survival stratified by ELN2017 risk groups.

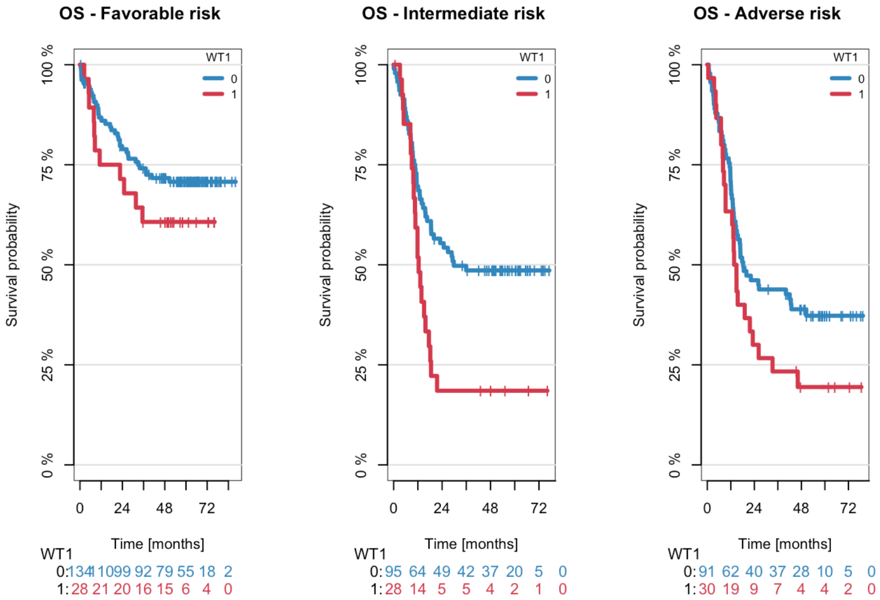

Supplemental Figure S5: Impact of *WT1* mutations on event-free survival stratified by ELN2017 risk groups.

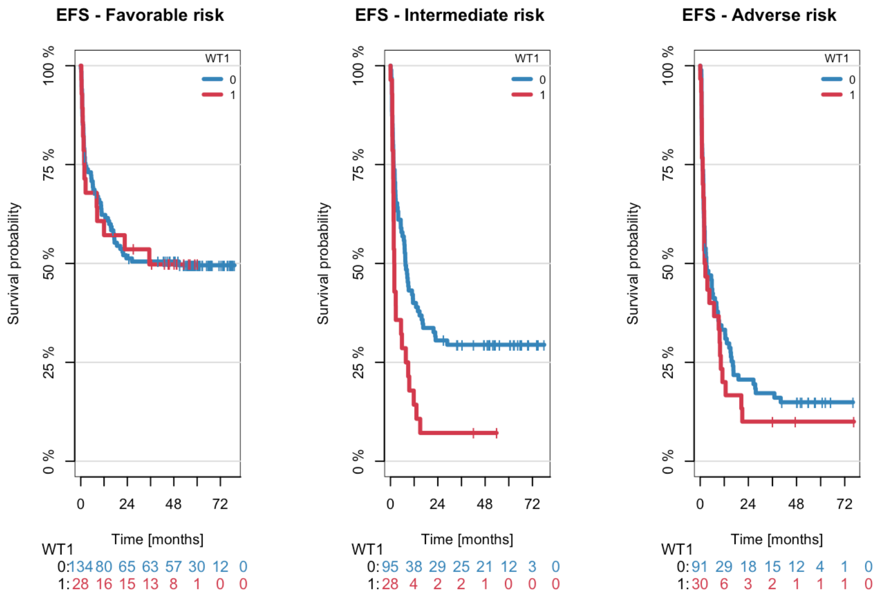

Supplemental Figure S6: Prognostic and possibly predictive impact of pairwise interactions of clinical and/or genetic variables in 475 patients on A) overall and B) event-free survival using random survival forests. The prognostic impact of a variable is measured via "vimp" (variable importance). This measure determines the loss in prediction accuracy using a permuted/noisy version of each variable for model fitting and predicting out-of-bag samples. The assessment of pairwise interactions between variables was based on the comparison of the joint ('paired') VIMP to the sum of their individual VIMPs (called 'additive' importance). Fitting 1000 trees per forest, we constructed 100 forests with different seeds. The following graphic depicts the variable importance for all varibles across these 100 runs in terms of variability (box plots). Higher positive or negative difference between additive and paired vimp values indicate that a variable combination may have prognostic or predictive impact on the survival endpoint.

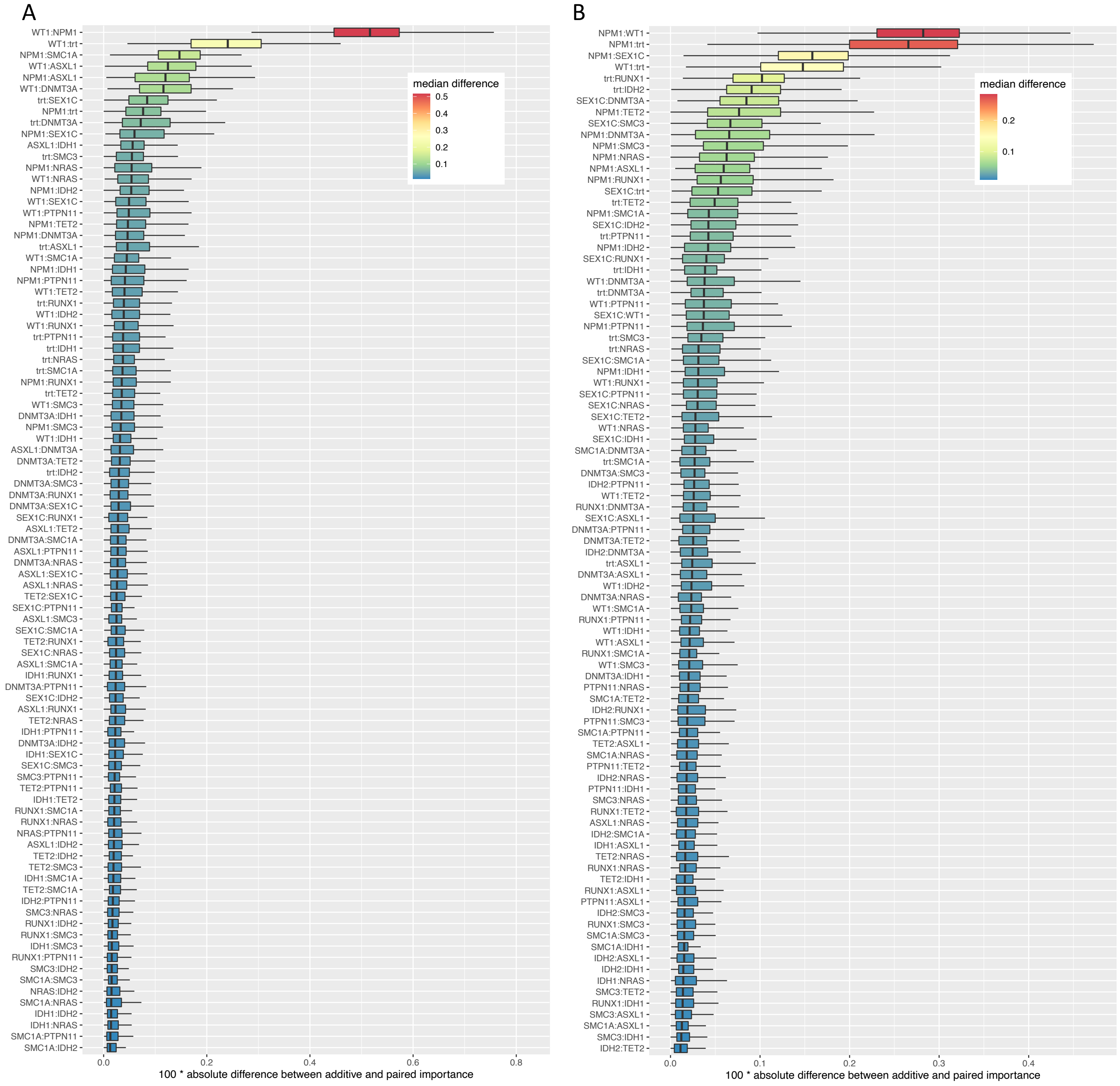

Supplemental Figure S7: The top 10 most interesting interactions regarding overall survival as determined by random survival forests were selected for further inspection. The following Kaplan Meier curves depict the marginal distribution of first variable (left) and the second variable (middle) as well as the combination of the two (right).

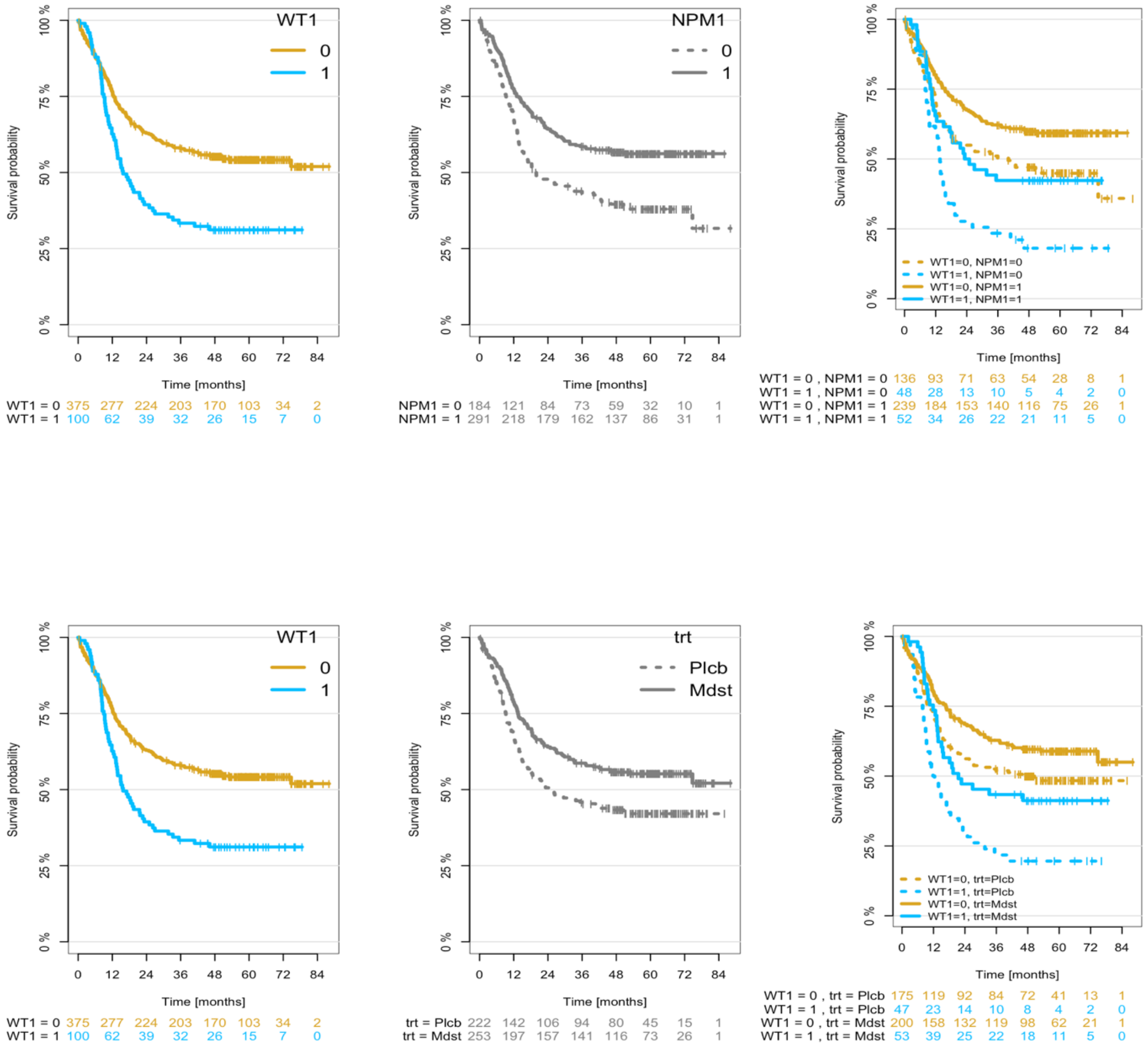

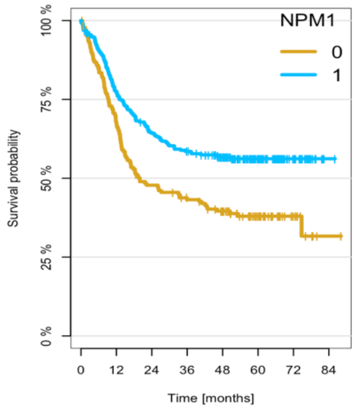

	0	12	24	36	48	60	72	84
NPM1 = 0	184	121	84	73	59	32	10	1
NPM1 = 1	291	218	179	162	137	86	31	1

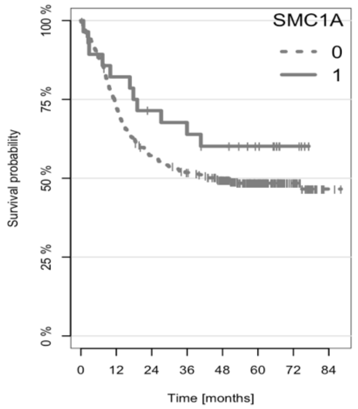

	0	12	24	36	48	60	72	84
SMC1A = 0	447	316	244	218	181	107	37	2
SMC1A = 1	28	23	19	17	15	11	4	0

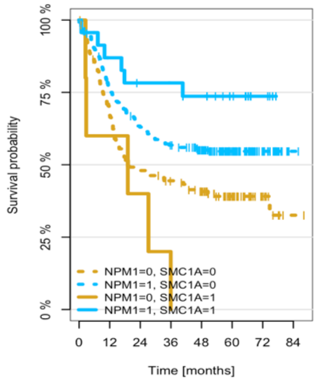

	0	12	24	36	48	60	72	84
NPM1 = 0 , SMC1A = 0	179	118	82	73	59	32	10	1
NPM1 = 1 , SMC1A = 0	268	198	162	145	122	75	27	1
NPM1 = 0 , SMC1A = 1	5	3	2	0	0	0	0	0
NPM1 = 1 , SMC1A = 1	23	20	17	17	15	11	4	0

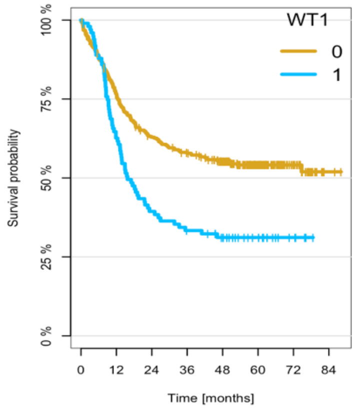

	0	12	24	36	48	60	72	84
WT1 = 0	375	277	224	203	170	103	34	2
WT1 = 1	100	62	39	32	26	15	7	0

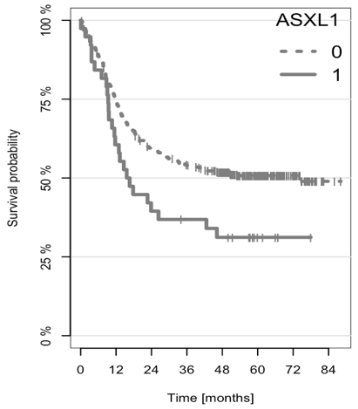

	0	12	24	36	48	60	72	84
ASXL1 = 0	437	316	248	222	185	114	40	2
ASXL1 = 1	38	23	15	13	11	4	1	0

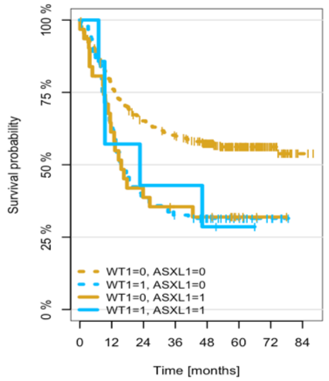

	0	12	24	36	48	60	72	84
WT1 = 0 , ASXL1 = 0	344	258	212	193	161	100	33	2
WT1 = 1 , ASXL1 = 0	93	58	36	29	24	14	7	0
WT1 = 0 , ASXL1 = 1	31	19	12	10	9	3	1	0
WT1 = 1 , ASXL1 = 1	7	4	3	3	2	1	0	0

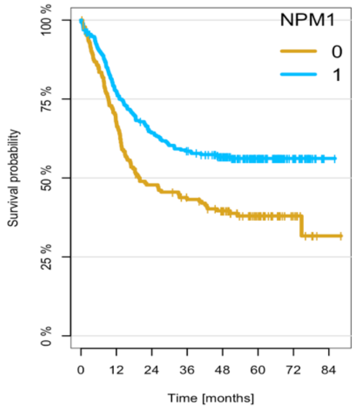

	0	12	24	36	48	60	72	84
NPM1 = 0	184	121	84	73	59	32	10	1
NPM1 = 1	291	218	179	162	137	86	31	1

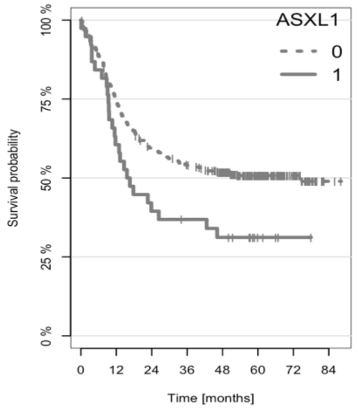

	0	12	24	36	48	60	72	84
ASXL1 = 0	437	316	248	222	185	114	40	2
ASXL1 = 1	38	23	15	13	11	4	1	0

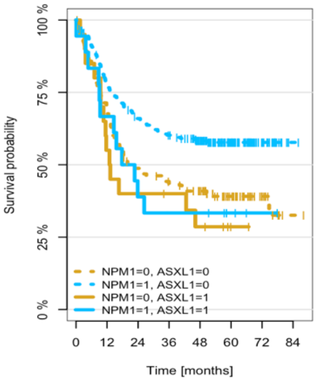

	0	12	24	36	48	60	72	84
NPM1 = 0 , ASXL1 = 0	164	110	76	66	54	31	10	1
NPM1 = 1 , ASXL1 = 0	273	206	172	156	131	83	30	1
NPM1 = 0 , ASXL1 = 1	20	11	8	7	5	1	0	0
NPM1 = 1 , ASXL1 = 1	18	12	7	6	6	3	1	0

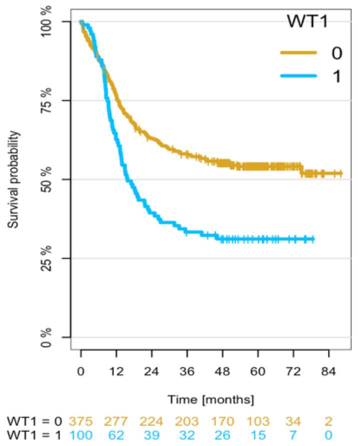
WT1
0
1
Survival probability
Time [months]
WT1 = 0 375 277 224 203 170 103 34 2
WT1 = 1 100 62 39 32 26 15 7 0

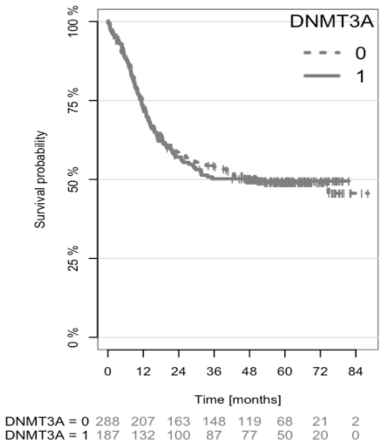
DNMT3A
0
1
Survival probability
Time [months]
DNMT3A = 0 288 207 163 148 119 68 21 2
DNMT3A = 1 187 132 100 87 77 50 20 0

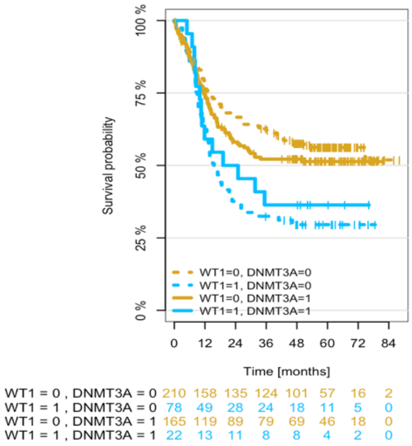
WT1=0, DNMT3A=0
WT1=1, DNMT3A=0
WT1=0, DNMT3A=1
WT1=1, DNMT3A=1
Survival probability
Time [months]
WT1 = 0 , DNMT3A = 0 210 158 135 124 101 57 16 2
WT1 = 1 , DNMT3A = 0 78 49 28 24 18 11 5 0
WT1 = 0 , DNMT3A = 1 165 119 89 79 69 46 18 0
WT1 = 1 , DNMT3A = 1 22 13 11 8 8 4 2 0

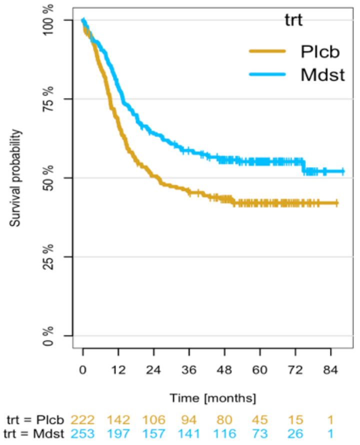
trt
Plcb
Mdst
Survival probability
Time [months]
trt = Plcb 222 142 106 94 80 45 15 1
trt = Mdst 253 197 157 141 116 73 26 1

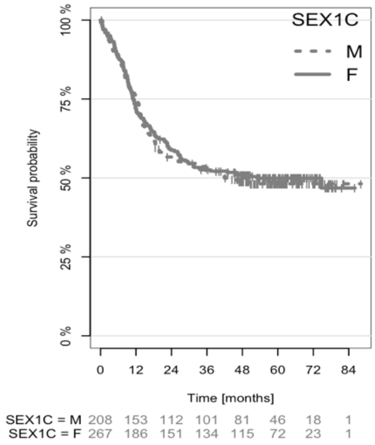
SEX1C
M
F
Survival probability
Time [months]
SEX1C = M 208 153 112 101 81 46 18 1
SEX1C = F 267 186 151 134 115 72 23 1

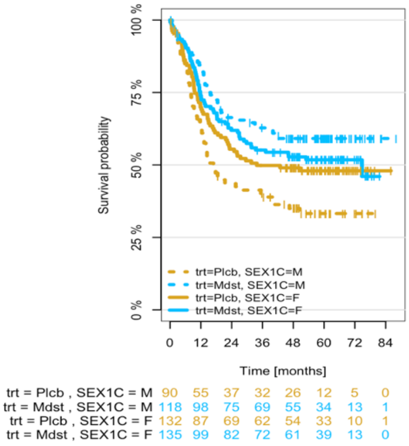
trt=Plcb, SEX1C=M
trt=Mdst, SEX1C=M
trt=Plcb, SEX1C=F
trt=Mdst, SEX1C=F
Survival probability
Time [months]
trt = Plcb , SEX1C = M 90 55 37 32 26 12 5 0
trt = Mdst , SEX1C = M 118 98 75 69 55 34 13 1
trt = Plcb , SEX1C = F 132 87 69 62 54 33 10 1
trt = Mdst , SEX1C = F 135 99 82 72 61 39 13 0

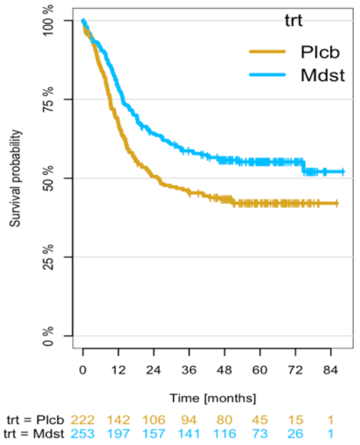
trt
Plcb
Mdst
Survival probability
Time [months]
trt = Plcb 222 142 106 94 80 45 15 1
trt = Mdst 253 197 157 141 116 73 26 1

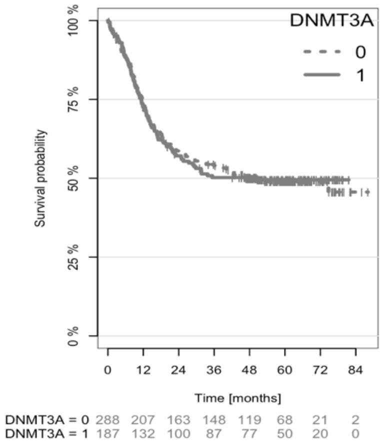
DNMT3A
0
1
Survival probability
Time [months]
DNMT3A = 0 288 207 163 148 119 68 21 2
DNMT3A = 1 187 132 100 87 77 50 20 0

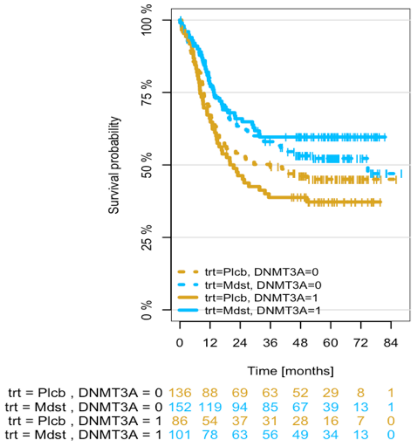
trt=Plcb, DNMT3A=0
trt=Mdst, DNMT3A=0
trt=Plcb, DNMT3A=1
trt=Mdst, DNMT3A=1
Survival probability
Time [months]
trt = Plcb , DNMT3A = 0 136 88 69 63 52 29 8 1
trt = Mdst , DNMT3A = 0 152 119 94 85 67 39 13 1
trt = Plcb , DNMT3A = 1 86 54 37 31 28 16 7 0
trt = Mdst , DNMT3A = 1 101 78 63 56 49 34 13 0

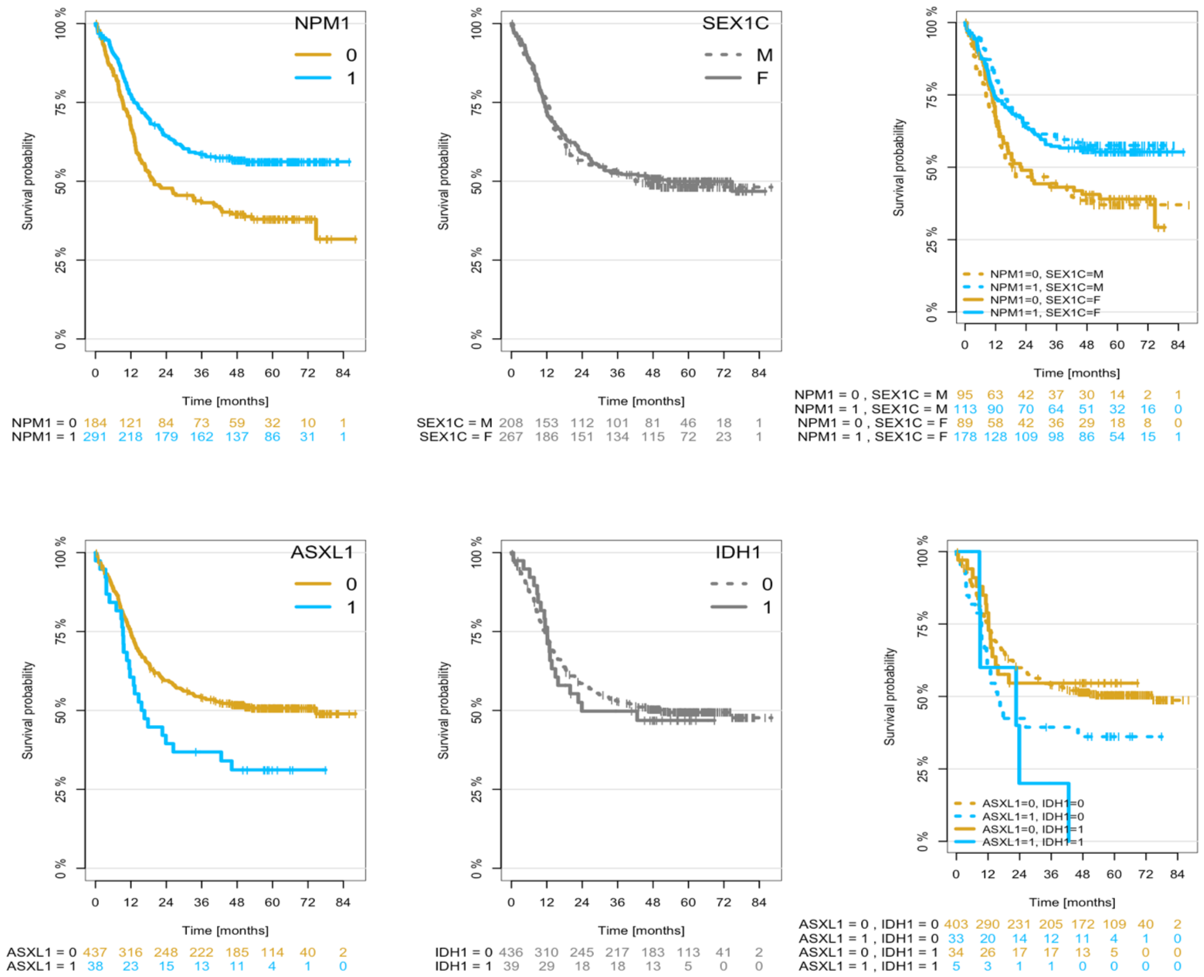
NPM1
0
1
Survival probability
Time [months]
NPM1 = 0 184 121 84 73 59 32 10 1
NPM1 = 1 291 218 179 162 137 86 31 1
SEX1C
M
F
SEX1C = M 208 153 112 101 81 46 18 1
SEX1C = F 267 186 151 134 115 72 23 1
NPM1=0, SEX1C=M
NPM1=1, SEX1C=M
NPM1=0, SEX1C=F
NPM1=1, SEX1C=F
NPM1 = 0 , SEX1C = M 95 63 42 37 30 14 2 1
NPM1 = 1 , SEX1C = M 113 90 70 64 51 32 16 0
NPM1 = 0 , SEX1C = F 89 58 42 36 29 18 8 0
NPM1 = 1 , SEX1C = F 178 128 109 98 86 54 15 1
ASXL1
ASXL1 = 0 437 316 248 222 185 114 40 2
ASXL1 = 1 38 23 15 13 11 4 1 0
IDH1
IDH1 = 0 436 310 245 217 183 113 41 2
IDH1 = 1 39 29 18 18 13 5 0 0
ASXL1=0, IDH1=0
ASXL1=1, IDH1=0
ASXL1=0, IDH1=1
ASXL1=1, IDH1=1
ASXL1 = 0 , IDH1 = 0 403 290 231 205 172 109 40 2
ASXL1 = 1 , IDH1 = 0 33 20 14 12 11 4 1 0
ASXL1 = 0 , IDH1 = 1 34 26 17 17 13 5 0 0
ASXL1 = 1 , IDH1 = 1 5 3 1 1 0 0 0 0

Supplemental Figure S8: The top 10 most interesting interactions regarding event-free survival as determined by random survival forests were selected for further inspection. The following Kaplan Meier curves depict the marginal distribution of first variable (left) and the second variable (middle) as well as the combination of the two (right).

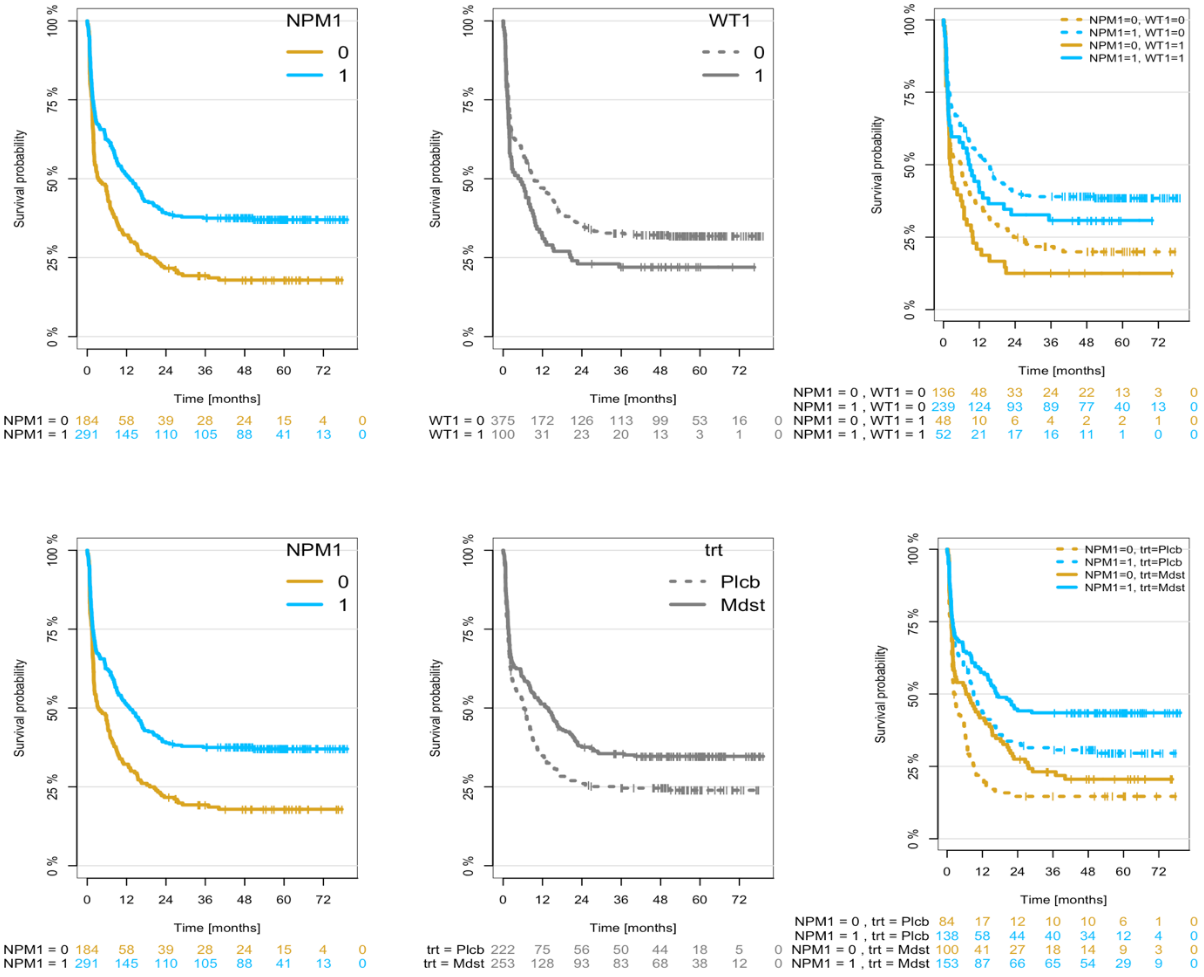

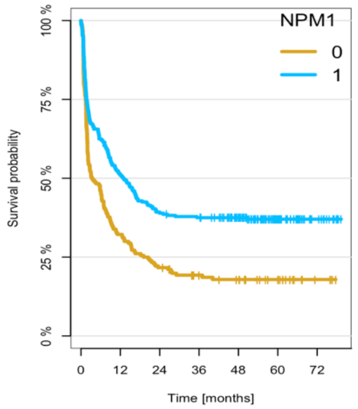

	0	12	24	36	48	60	72	
NPM1 = 0	184	58	39	28	24	15	4	0
NPM1 = 1	291	145	110	105	88	41	13	0

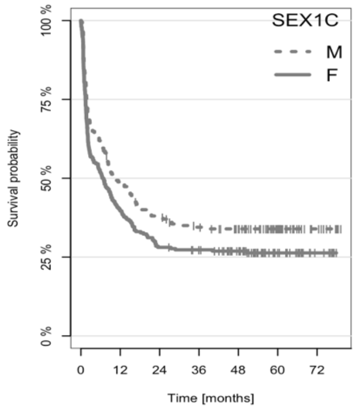

	0	12	24	36	48	60	72	
SEX1C = M	208	100	76	66	58	32	10	0
SEX1C = F	267	103	73	67	54	24	7	0

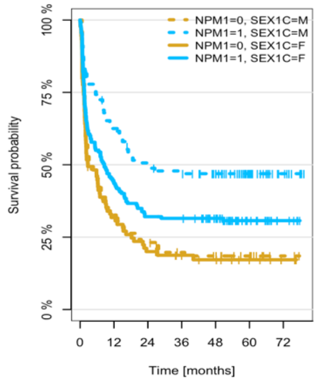

	0	12	24	36	48	60	72	
NPM1 = 0 , SEX1C = M	95	31	22	16	14	9	2	0
NPM1 = 1 , SEX1C = M	113	69	54	50	44	23	8	0
NPM1 = 0 , SEX1C = F	89	27	17	12	10	6	2	0
NPM1 = 1 , SEX1C = F	178	76	56	55	44	18	5	0

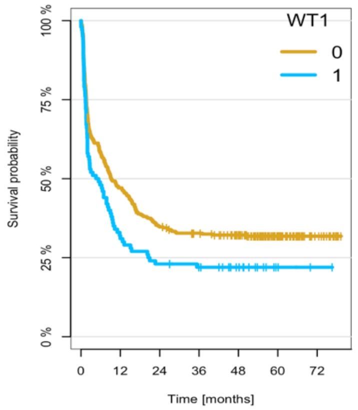

	0	12	24	36	48	60	72	
WT1 = 0	375	172	126	113	99	53	16	0
WT1 = 1	100	31	23	20	13	3	1	0

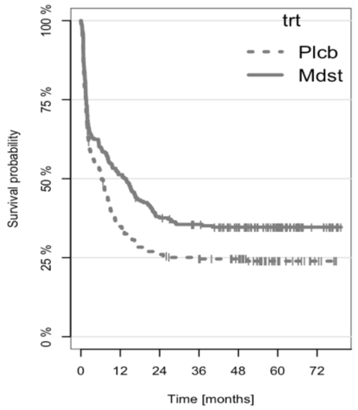

	0	12	24	36	48	60	72	
trt = Plcb	222	75	56	50	44	18	5	0
trt = Mdst	253	128	93	83	68	38	12	0

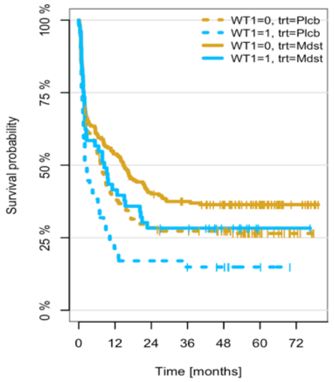

	0	12	24	36	48	60	72	
WT1 = 0 , trt = Plcb	175	66	48	44	39	16	5	0
WT1 = 1 , trt = Plcb	47	9	8	6	5	2	0	0
WT1 = 0 , trt = Mdst	200	106	78	69	60	37	11	0
WT1 = 1 , trt = Mdst	53	22	15	14	8	1	1	0

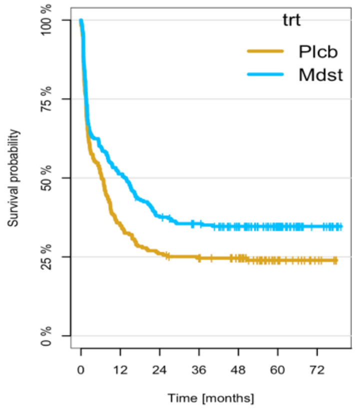

	0	12	24	36	48	60	72	
trt = Plcb	222	75	56	50	44	18	5	0
trt = Mdst	253	128	93	83	68	38	12	0

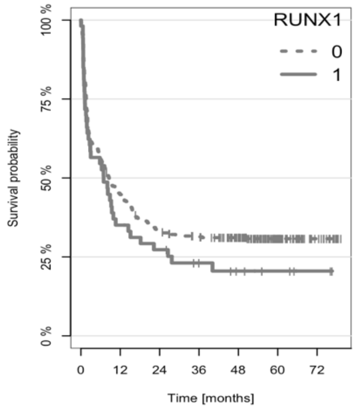

	0	12	24	36	48	60	72	
RUNX1 = 0	422	185	135	124	106	52	15	0
RUNX1 = 1	53	18	14	9	6	4	2	0

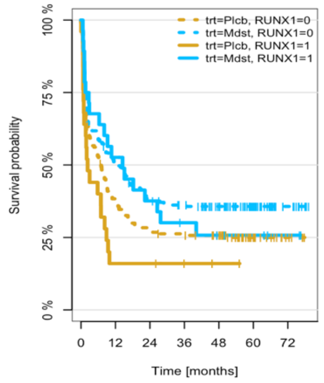

	0	12	24	36	48	60	72	
trt = Plcb , RUNX1 = 0	197	71	52	48	43	18	5	0
trt = Mdst , RUNX1 = 0	225	114	83	76	63	34	10	0
trt = Plcb , RUNX1 = 1	25	4	4	2	1	0	0	0
trt = Mdst , RUNX1 = 1	28	14	10	7	5	4	2	0

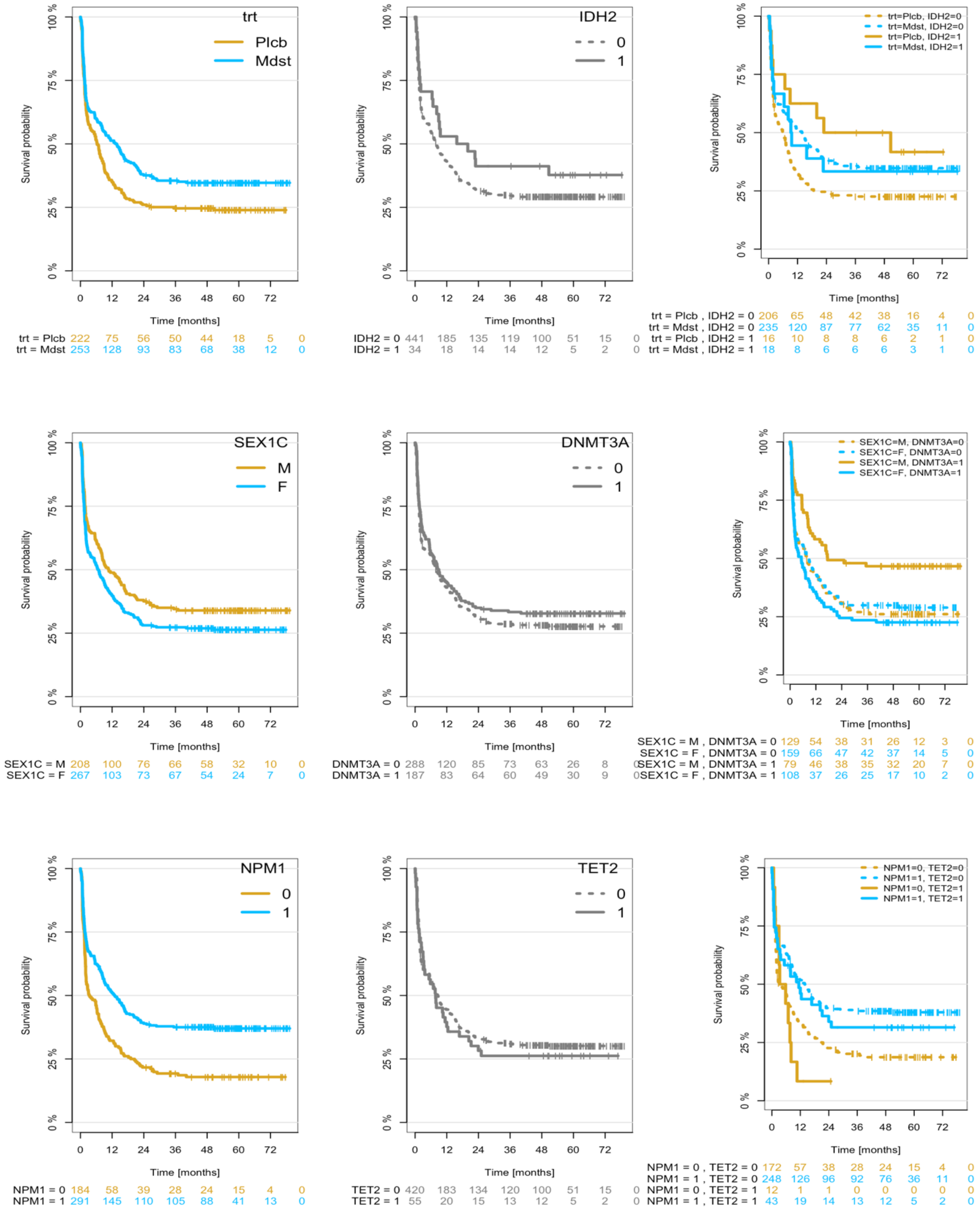
trt
Plcb
Mdst
Survival probability
Time [months]
trt = Plcb 222 75 56 50 44 18 5 0
trt = Mdst 253 128 93 83 68 38 12 0
IDH2
0
1
IDH2 = 0 441 185 135 119 100 51 15 0
IDH2 = 1 34 18 14 14 12 5 2 0
trt=Plcb, IDH2=0
trt=Mdst, IDH2=0
trt=Plcb, IDH2=1
trt=Mdst, IDH2=1
trt = Plcb , IDH2 = 0 206 65 48 42 38 16 4 0
trt = Mdst , IDH2 = 0 235 120 87 77 62 35 11 0
trt = Plcb , IDH2 = 1 16 10 8 8 6 2 1 0
trt = Mdst , IDH2 = 1 18 8 6 6 6 3 1 0
SEX1C
M
F
SEX1C = M 208 100 76 66 58 32 10 0
SEX1C = F 267 103 73 67 54 24 7 0
DNMT3A
0
1
DNMT3A = 0 288 120 85 73 63 26 8 0
DNMT3A = 1 187 83 64 60 49 30 9 0
SEX1C=M, DNMT3A=0
SEX1C=F, DNMT3A=0
SEX1C=M, DNMT3A=1
SEX1C=F, DNMT3A=1
SEX1C = M , DNMT3A = 0 129 54 38 31 26 12 3 0
SEX1C = F , DNMT3A = 0 159 66 47 42 37 14 5 0
SEX1C = M , DNMT3A = 1 79 46 38 35 32 20 7 0
SEX1C = F , DNMT3A = 1 108 37 26 25 17 10 2 0
NPM1
0
1
NPM1 = 0 184 58 39 28 24 15 4 0
NPM1 = 1 291 145 110 105 88 41 13 0
TET2
0
1
TET2 = 0 420 183 134 120 100 51 15 0
TET2 = 1 55 20 15 13 12 5 2 0
NPM1=0, TET2=0
NPM1=1, TET2=0
NPM1=0, TET2=1
NPM1=1, TET2=1
NPM1 = 0 , TET2 = 0 172 57 38 28 24 15 4 0
NPM1 = 1 , TET2 = 0 248 126 96 92 76 36 11 0
NPM1 = 0 , TET2 = 1 12 1 1 0 0 0 0 0
NPM1 = 1 , TET2 = 1 43 19 14 13 12 5 2 0

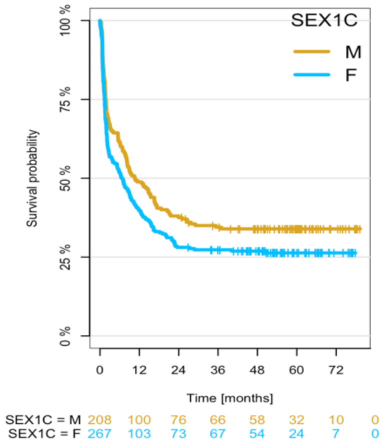
SEX1C
M
F
Survival probability
100 %
75 %
50 %
25 %
0 %
0 12 24 36 48 60 72
Time [months]
SEX1C = M 208 100 76 66 58 32 10 0
SEX1C = F 267 103 73 67 54 24 7 0

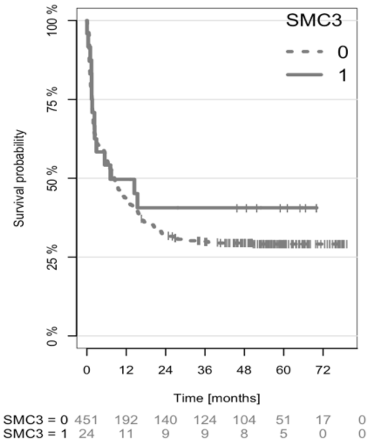
SMC3
0
1
Survival probability
100 %
75 %
50 %
25 %
0 %
0 12 24 36 48 60 72
Time [months]
SMC3 = 0 451 192 140 124 104 51 17 0
SMC3 = 1 24 11 9 9 8 5 0 0

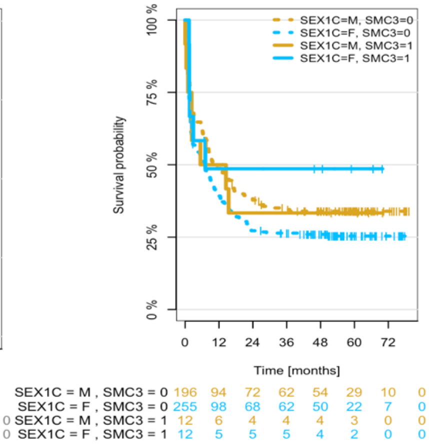
SEX1C=M, SMC3=0
SEX1C=F, SMC3=0
SEX1C=M, SMC3=1
SEX1C=F, SMC3=1
Survival probability
100 %
75 %
50 %
25 %
0 %
0 12 24 36 48 60 72
Time [months]
SEX1C = M , SMC3 = 0 196 94 72 62 54 29 10 0
SEX1C = F , SMC3 = 0 255 98 68 62 50 22 7 0
SEX1C = M , SMC3 = 1 12 6 4 4 4 3 0 0
SEX1C = F , SMC3 = 1 12 5 5 5 4 2 0 0

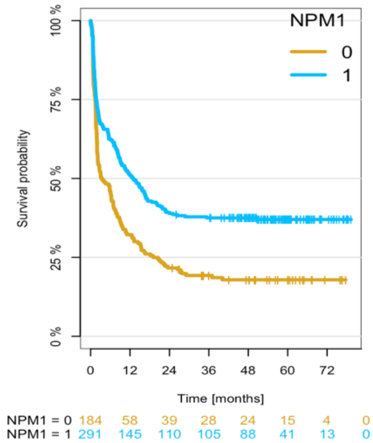
NPM1
0
1
Survival probability
100 %
75 %
50 %
25 %
0 %
0 12 24 36 48 60 72
Time [months]
NPM1 = 0 184 58 39 28 24 15 4 0
NPM1 = 1 291 145 110 105 88 41 13 0

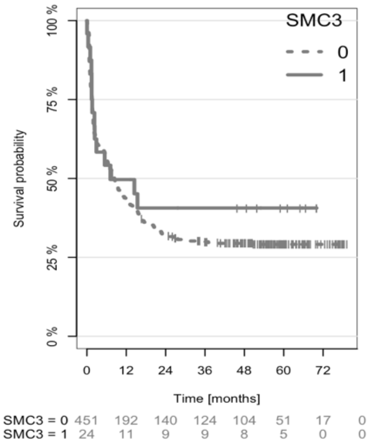
SMC3
0
1
Survival probability
100 %
75 %
50 %
25 %
0 %
0 12 24 36 48 60 72
Time [months]
SMC3 = 0 451 192 140 124 104 51 17 0
SMC3 = 1 24 11 9 9 8 5 0 0

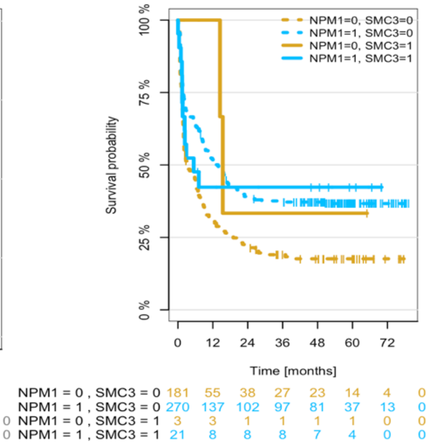
NPM1=0, SMC3=0
NPM1=1, SMC3=0
NPM1=0, SMC3=1
NPM1=1, SMC3=1
Survival probability
100 %
75 %
50 %
25 %
0 %
0 12 24 36 48 60 72
Time [months]
NPM1 = 0 , SMC3 = 0 181 55 38 27 23 14 4 0
NPM1 = 1 , SMC3 = 0 270 137 102 97 81 37 13 0
NPM1 = 0 , SMC3 = 1 3 3 1 1 1 1 0 0
NPM1 = 1 , SMC3 = 1 21 8 8 8 7 4 0 0

Supplemental Figure S9: Kaplan Meier estimates of impact of *NPM1*, *DNMT3A*, and combined genotypes on overall (A) and event-free (B) survival and according to *FLT3* mutation type.

A

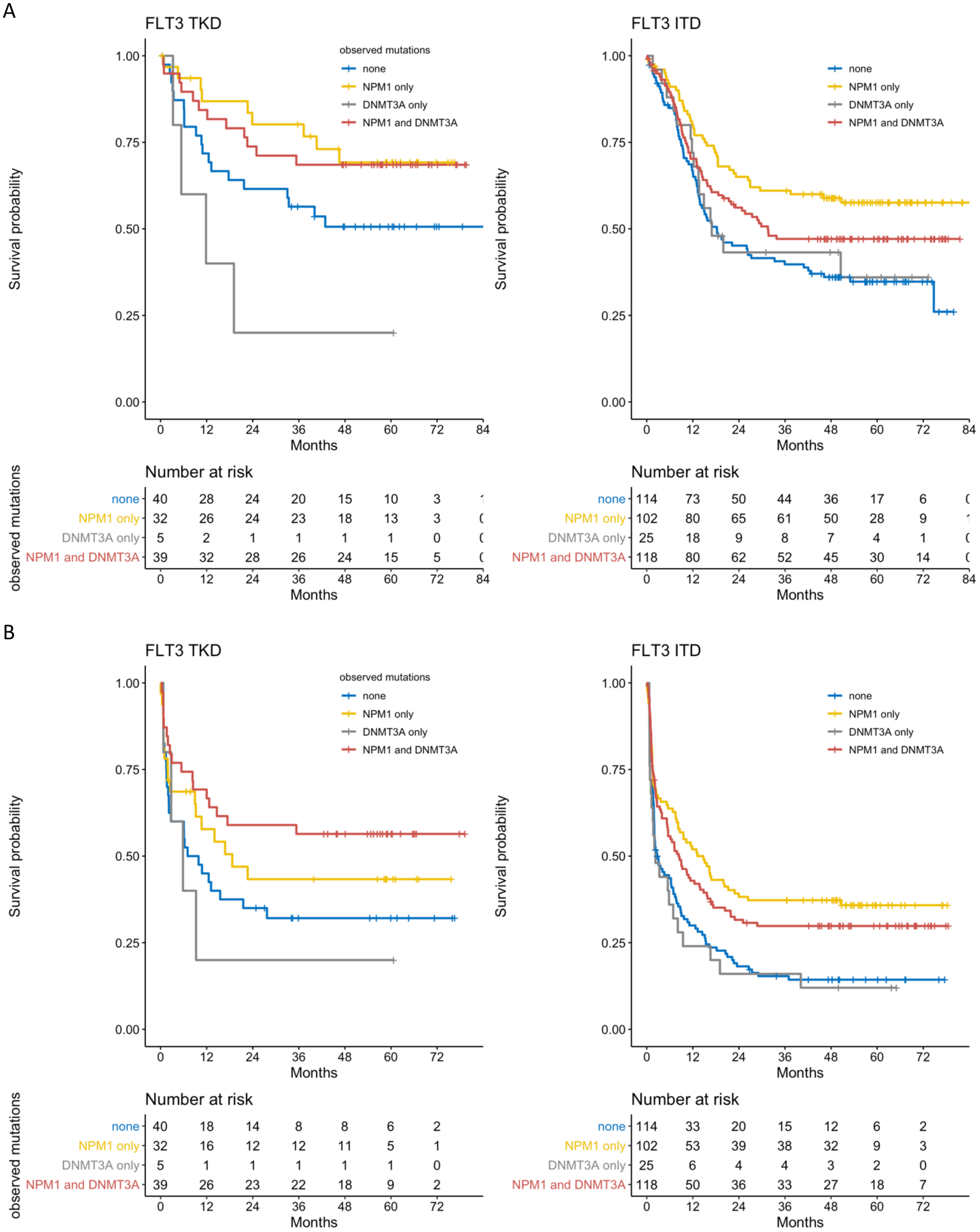

Supplemental Figure S10: Kaplan-Meier estimated A) overall and B) event-free survival curves, and number of events by genomic AML classes and log rank test p-values in cohort of 451 of 475 patients, in which subcategorization into genomic AML classes was possible. Abbreviations: OS, overall survival; EFS, event-free survival.

A

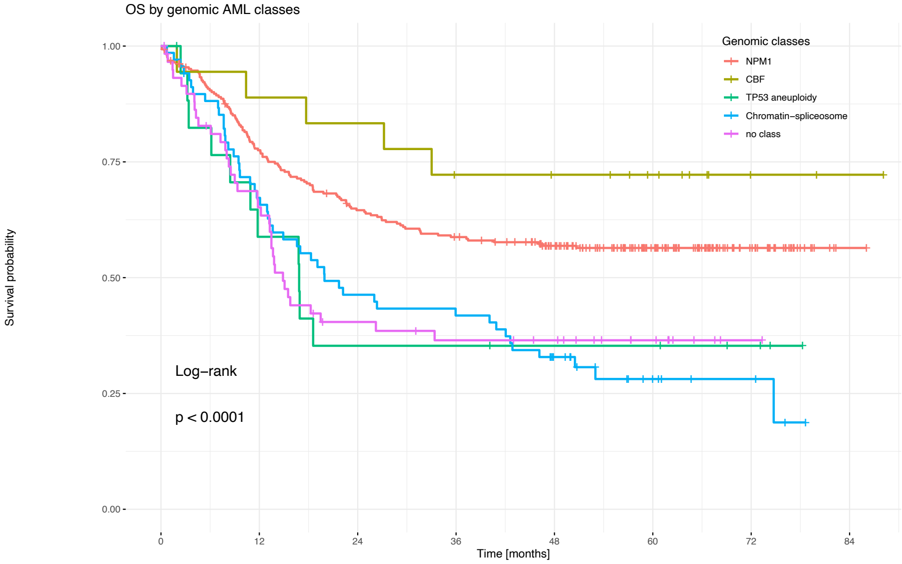

OS by genomic AML classes

Genomic classes	0	12	24	36	48	60	72	84
NPM1	287	216	178	161	136	85	31	1
CBF	18	16	15	12	11	8	2	1
TP53 aneuploidy	18	10	6	6	5	5	3	0
Chromatin−spliceosome	68	45	31	28	18	7	4	0
no class	60	37	21	18	16	9	1	0

Time [months]

B

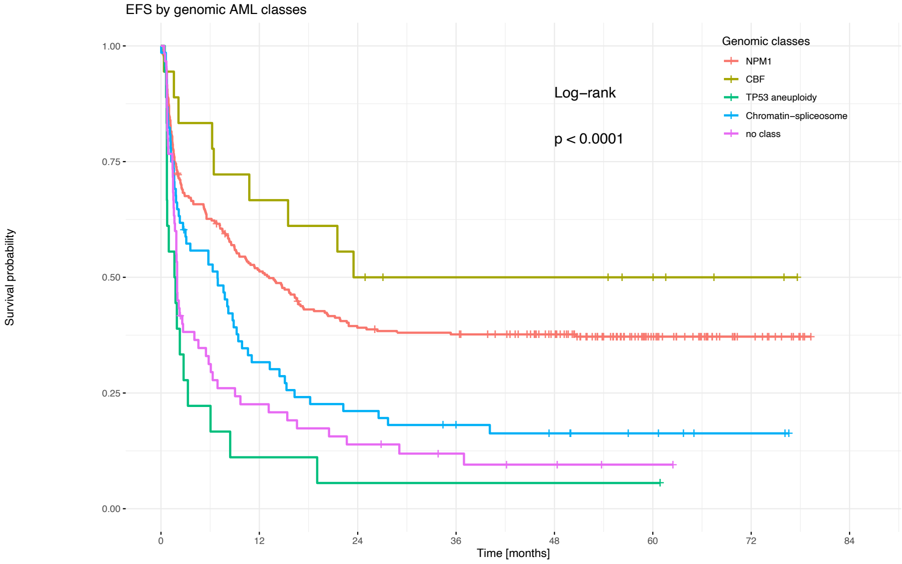

EFS by genomic AML classes

Genomic classes	0	12	24	36	48	60	72	84
NPM1	287	144	109	104	87	40	13	0
CBF	18	12	9	7	7	5	2	0
TP53 aneuploidy	18	2	1	1	1	1	0	0
Chromatin−spliceosome	68	21	14	10	8	5	2	0
no class	60	13	8	5	3	1	0	0

Time [months]

Supplemental Figure S11: Kaplan-Meier plots for the marginal overall survival (OS) distribution in the corresponding genomic AML classes (pCat) and treatment (trt) subgroups. Abbreviations: C-S, chromatin-spliceosome; Mdst, midostaurin; Plcb, placebo; TP53an, TP53-aneuploidy.

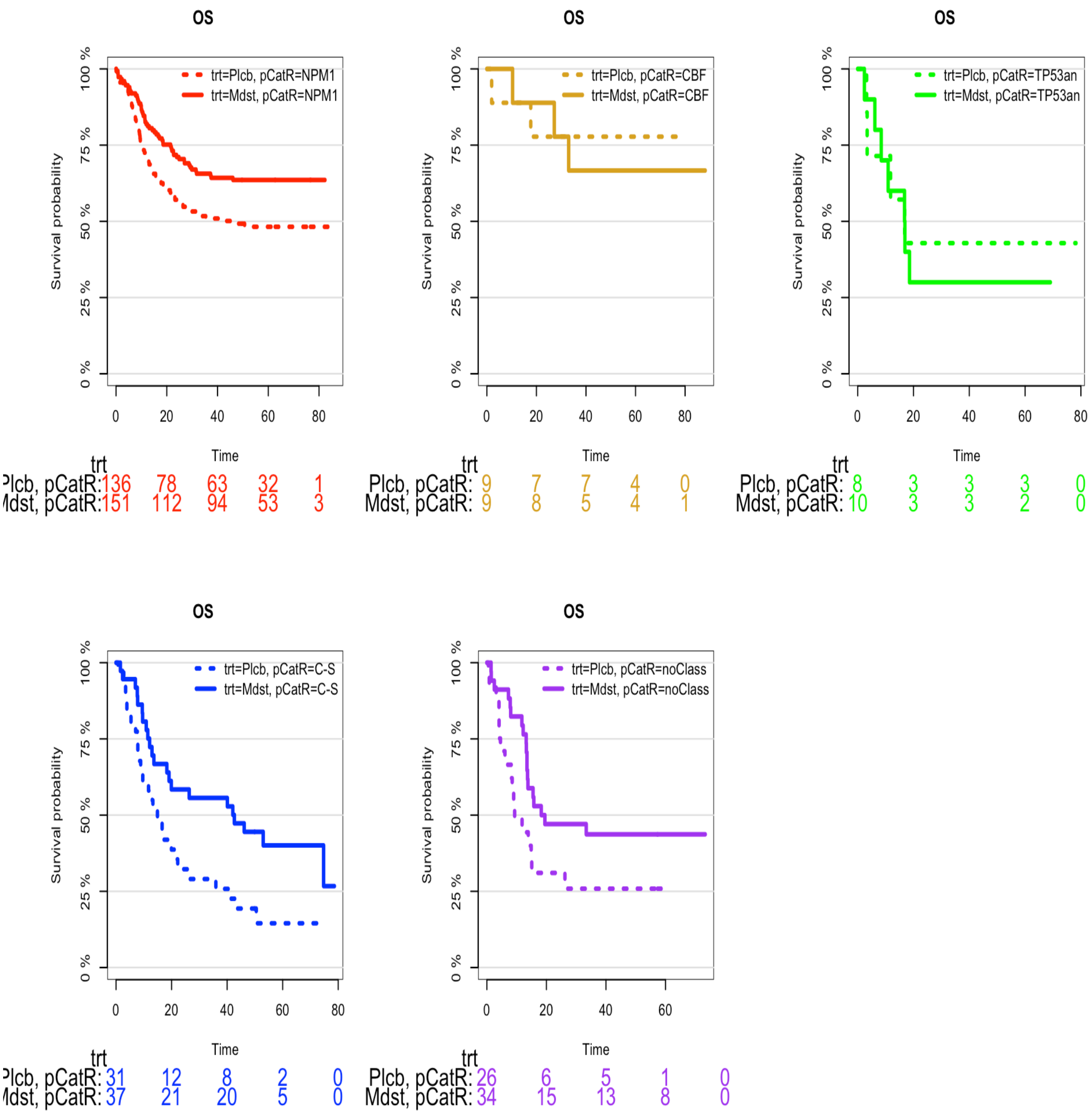

Supplemental Figure S12: Kaplan-Meier plots for the marginal event-free survival (EFS) distribution in the corresponding genomic AML classes (pCat) and treatment (trt) subgroups. Abbreviations: C-S, chromatin-spliceosome; Mdst, midostaurin; Plcb, placebo; TP53an, TP53-aneuploidy.

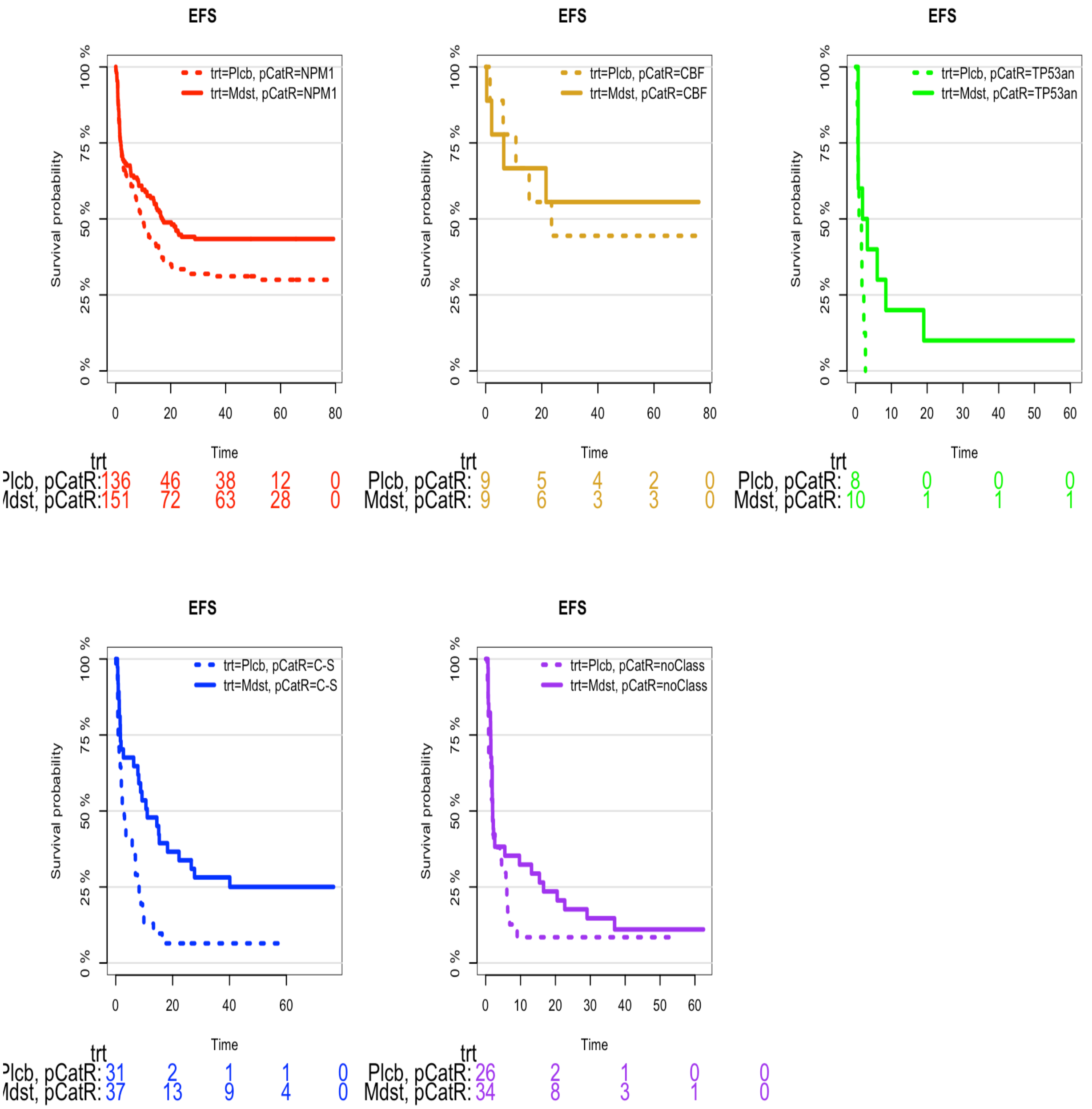

Supplemental Figure S13: Kaplan-Meier estimates for A) overall and B) event-free survival according to *FLT3* mutation type and treatment of 451 patients included into the Cox proportional hazard model.

A

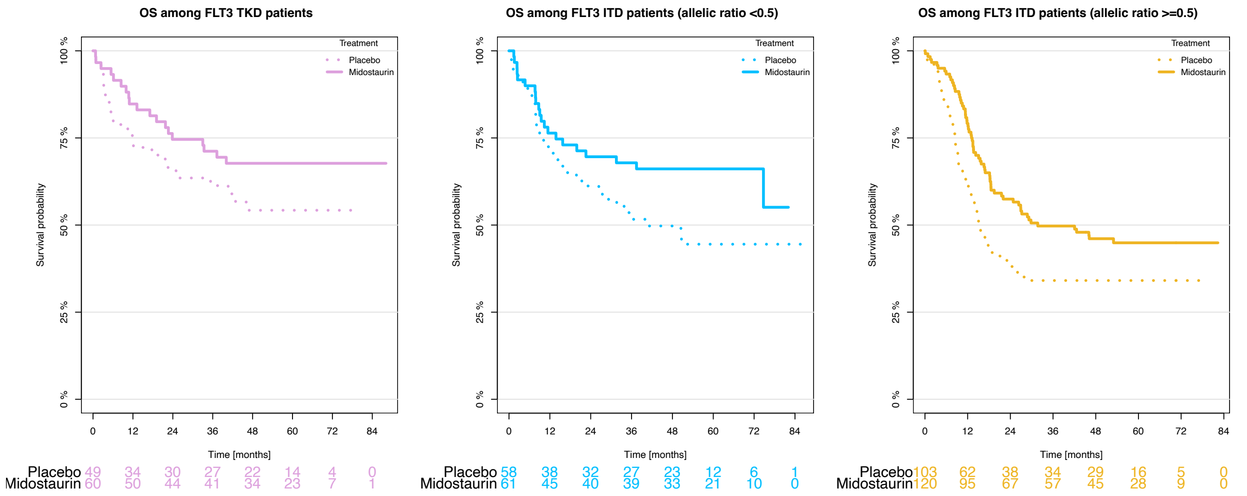

B

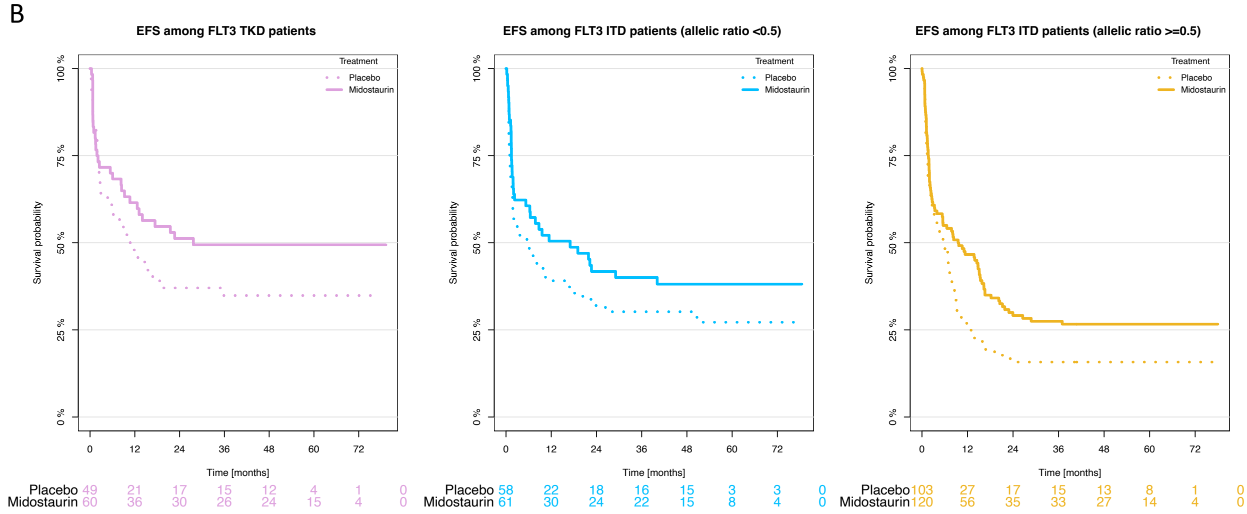

Supplemental Figure S14: Kaplan Meier estimates for A) overall and C) event-free survival by midostaurin kinome mutation status in entire cohort. Effect of treatment on B) overall and D) event-free survival in subgroup of patients harboring midostaurin kinome mutations.

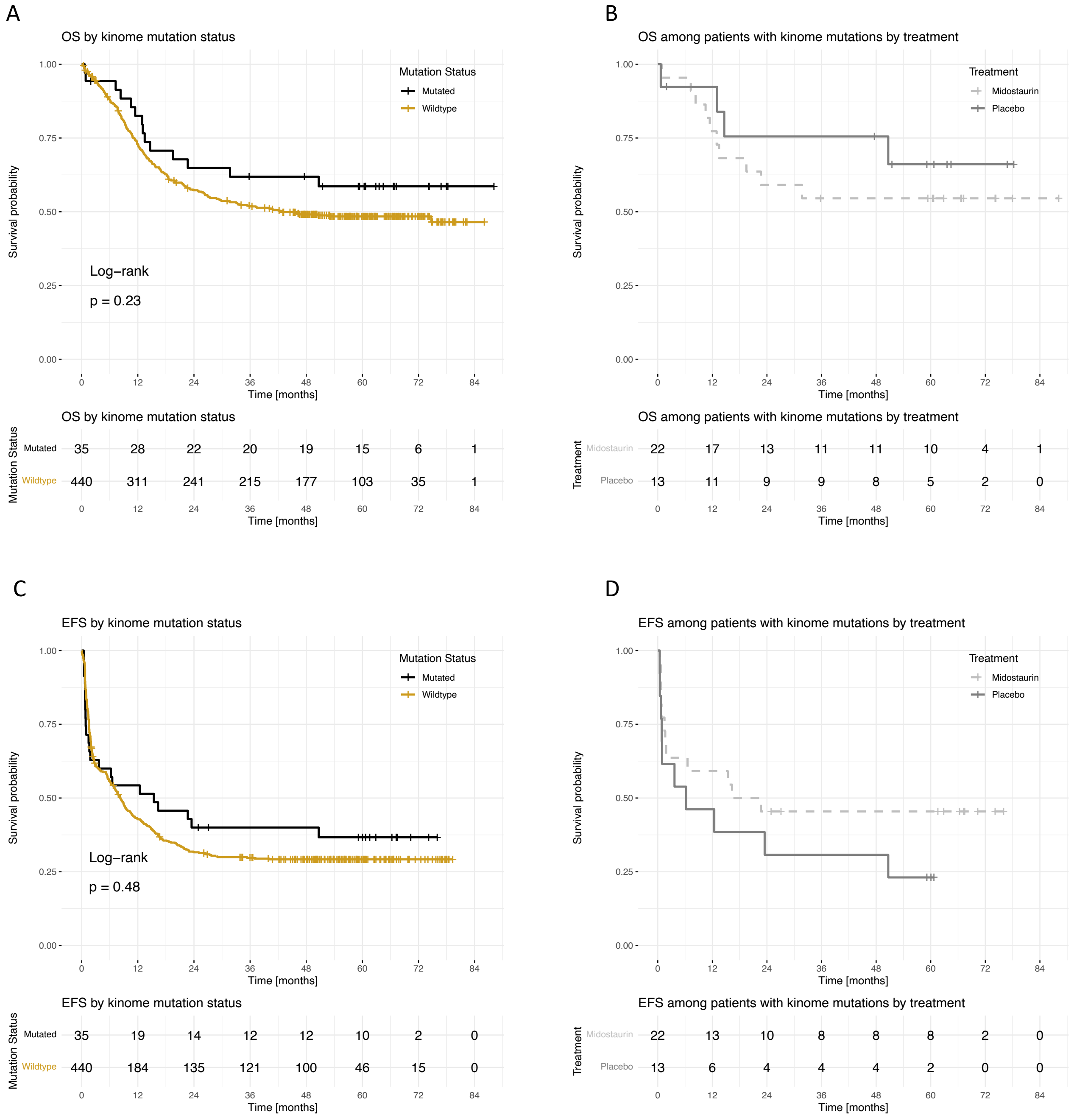

Supplemental Figure S15: Kaplan Meier estimator for overall (OS) and event-free survival (EFS) according to *FLT3* exon 16 mutation status.

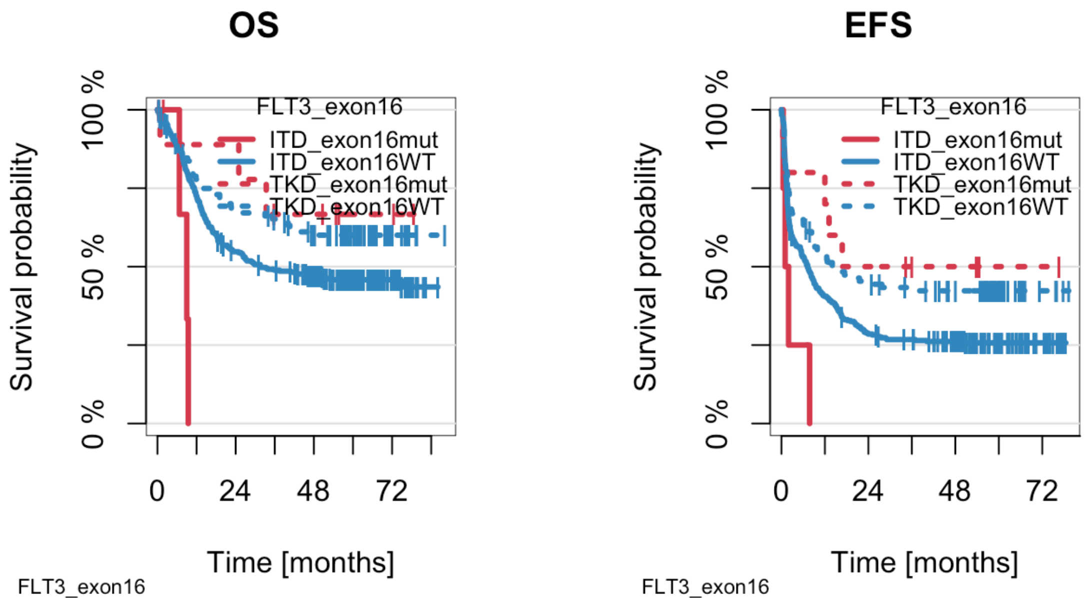

OS, FLT3_exon16:

ITD_exon16mut:	4	0	0	0	0	0	0	0
ITD_exon16WT:	355	251	186	165	138	79	30	1
TKD_exon16mut:	10	8	8	5	5	2	2	0
TKD_exon16WT:	106	80	69	65	53	37	9	1

EFS, FLT3_exon16:

ITD_exon16mut:	4	0	0	0	0	0	0	0
ITD_exon16WT:	355	142	99	90	74	35	12	0
TKD_exon16mut:	10	7	5	3	3	1	1	0
TKD_exon16WT:	106	54	45	40	35	20	4	0
